# Non-cytotoxic Cardiac Innate Lymphoid Cells Are a Resident and Quiescent Type 2-Commited Population

**DOI:** 10.3389/fimmu.2019.00634

**Published:** 2019-03-29

**Authors:** William Bracamonte-Baran, Guobao Chen, Xuezhou Hou, Monica V. Talor, Hee Sun Choi, Giovanni Davogustto, Heinrich Taegtmeyer, Jungeun Sung, David Joel Hackam, David Nauen, Daniela Čiháková

**Affiliations:** ^1^Department of Pathology, School of Medicine, Johns Hopkins University, Baltimore, MD, United States; ^2^W. Harry Feinstone Department of Molecular Microbiology and Immunology, Bloomberg School of Public Health, Johns Hopkins University, Baltimore, MD, United States; ^3^Division of Cardiology, Department of Internal Medicine, University of Texas Medical School at Houston, Houston, TX, United States; ^4^School of Medicine, Institute of Genetic Medicine, Johns Hopkins University, Baltimore, MD, United States; ^5^Division of General Pediatric Surgery, Johns Hopkins University and Bloomberg Children's Center, Johns Hopkins Hospital, Baltimore, MD, United States

**Keywords:** innate lymphoid cells, IL-33, heart, myocarditis, myocardial infarction, fibroblasts

## Abstract

Innate lymphoid cells (ILC) are a subset of leukocytes with lymphoid properties that lack antigen specific receptors. They can be stimulated by and exert their effect via specific cytokine axes, whereas Natural Killers (NK) cells are the only known cytotoxic member of this family. ILCs are considered key in linking the innate and adaptive response in physiologic and pathologic environments. In this study, we investigated the properties of non-cytotoxic cardiac ILCs in physiologic, inflammatory, and ischemic conditions. We found that in healthy humans and mice, non-cytotoxic cardiac ILCs are predominantly a type 2-committed population with progenitor-like features, such as an absence of type-specific immunophenotype, intermediate GATA3 expression, and capacity to transiently express Pro-myelocytic Leukemia Zinc Finger protein (PLZF) upon activation. During myocarditis and ischemia, in both human and mice, cardiac ILCs differentiated into conventional ILC2s. We found that cardiac ILCs lack IL-25 receptor and cannot become inflammatory ILC2s. We found a strong correlation between IL-33 production in the heart and the ability of cardiac ILCs to become conventional ILC2s. The main producer of IL-33 was a subset of CD29+Sca-1+ cardiac fibroblasts. ILC2 expansion and fibroblast-derived IL-33 production were significantly increased in the heart in mouse models of infarction and myocarditis. Despite its progenitor-like status in healthy hearts, cardiac ILCs were unable to become ILC1 or ILC3 *in vivo* and *in vitro*. Using adoptive transfer and parabiosis, we demonstrated that the heart, unlike other organs such as lung, cannot be infiltrated by circulating ILCs in adulthood even during cardiac inflammation or ischemia. Thus, the ILC2s present during inflammatory conditions are derived from the heart-resident and quiescent steady-state population. Non-cytotoxic cardiac ILCs are a resident population of ILC2-commited cells, with undifferentiated progenitor-like features in steady-state conditions and an ability to expand and develop pro-inflammatory type 2 features during inflammation or ischemia.

## Introduction

Innate lymphoid cells (ILCs), formerly named nuocytes (1), are a subset of leukocytes with lymphoid properties in terms of their responsiveness and cytokine production, but lack antigen specific receptors (2–4). ILCs are considered to play a key role in the cross-talk between the innate and adaptive immune responses, thus providing a functional link between them (5).

Three classic subsets of ILCs have been described, named ILCs type 1 (ILC1s), type 2 (ILC2s), and type 3 (ILC3s), based on their functional profiles (3). ILCs plasticity and capacity of trans-differentiation have been widely reported (6–8). Non-cytotoxic ILC1s mirror and complement Th1 response, as those cells are stimulated by IL-12, and express IFNγ and TNFα upon activation (9, 10). ILC1s development depends on the transcription nuclear factor Tbet (11). The main role of ILC1's is assisting in the anti-viral response in a non-cytotoxic manner, thus supporting the Th1 response (12–14). Natural Killer (NK) cells are considered the only known cytotoxic subtype of ILCs, belong to the type 1 subset, and display significant functional and phenotypic differences as compared with non-cytotoxic ILCs (12).

ILC2s are classic mediators of the Th2 response and have been associated with typical Th2 processes, like anti-helmintic and allergic responses in the gastrointestinal and respiratory tracts (15–19). In addition, ILC2s were shown to be involved in tissue remodeling in the lung and liver and also play a role in regulation of the lipid metabolism in adipose tissues (20–24). The main products of ILC2s are IL-5 and IL-13, which contribute to eosinophils and other granulocytes maturation in the bone marrow and their chemotaxis. ILC2s characteristic nuclear transcription factor is GATA3 (17, 25–28). The main stimulator of classic ILC2s is IL-33, a cytokine known to be produced as an alarmin by a wide range of cells, such as endothelial cells during cellular stress and noxious conditions (29–32). In lung tissues, a subset of inflammatory ILC2s (i-ILC2s) was described that specifically responds to IL-25 stimulation but not IL-33 and has a combination of ILC2 and ILC3-like properties (33–35).

ILC3s contribute to the Th17 response by producing IL-17A and GM-CSF in response to IL-23 and IL-1β stimulation (36, 37). ILC3s differentiation is dependent on the nuclear factor Rorγt (38). This ILC subset is involved in anti-bacteria responses but is also linked to autoimmune diseases such as psoriasis, inflammatory bowel disease, and ankylosing spondylitis (39–41).

ILCs originate in the bone marrow from common lymphoid progenitors that give rise to common helper innate lymphoid progenitors (CHILP), which progress to ILCs progenitors (ILCPs) (25, 42, 43). ILCPs finally differentiate into classic ILC1s, ILC2s, or ILC3s, depending on the specific cytokine milieu in the target organ (25). Moreover, several stages of differentiation were described between ILCPs and classically differentiated ILCs. A first example are ILC2 progenitors, which have been found predominantly in the bone marrow, but also described in murine liver tissues (44). ILC2 progenitors have been described to have a Lineage^neg^Sca-1^+^Id2^+^GATA3^+^ phenotype, with upregulation of ILC type 2-associated genes as *Klrg1, Il2r*α*, Ccr9* (27, 44–46). The second example are peripheral human multipotent ILCPs, which lack type-specific phenotype but express CD117 (cKit) (47). Peripheral ILCPs are a circulating population that has been described as being able to infiltrate organs such as liver, lung, and cord blood, and its final fate is determined by tissue-specific microenvironments, being able to differentiate into ILC1s, ILC2s, and ILC3s (47). The development of ILCs depends on the expression of the IL-2 receptor common γ-chain (γc), whereas recombinant activating gene (RAG) is not required (48). GATA3, although considered characteristic of fully differentiated classic ILC2s, is also required for the development of ILCPs (49). In addition, a nuclear factor—the Pro-myelocytic Leukemia Zinc Finger Protein (PLZF)—is needed for the development of ILCPs and its differentiation into specific ILC types. PLZF is known to be transiently expressed during ILCP activation and differentiation (42, 49). Although it was reported that PLZF gene expression (*Zbtb16*) is transversally decreased in ILC2 progenitors as compared to earlier progenitors (44), its complex expression dynamics over time during ILC development has not been completely elucidated.

Little is known about non-cytotoxic cardiac ILCs. Their identity, origin, phenotype, and functionality has not been studied so far. We have previously described the role NK cells in myocarditis (50, 51). Thus, in this study we seek to comprehensively characterize non-cytotoxic cardiac ILCs, their function and behavior during heart inflammatory diseases.

Compared with other organs in which ILCs have been described, the heart has unique histological, cellular, and functional properties. Volumetrically, the heart is mainly a muscular organ, lacking a classic epithelium, but rather having an extensive endothelial layer in the endocardium and a serosal epithelium, which constitute the pericardium (52). Multiple type of mesenchymal and bone marrow-derived cells reside in the heart, generating a complex microenvironment (53). The lineage and origins of cardiac cells are complex and not entirely understood (52). We and others have shown that cardiac fibroblasts are active contributors to cardiac inflammation by producing GM-CSF, CCL2, or CCL11 (54–58). Interestingly, IL-33 is produced by cardiac endothelial cells during pressure overload (30).

In this study, we evaluated non-cytotoxic cardiac ILCs in healthy human and mouse hearts and in ischemic cardiomyopathy and myocarditis. We found that cardiac ILCs are an ILC2-commited population. Under normal conditions, the cardiac ILC population was mainly undifferentiated with an incomplete ILC2 phenotype, while lacking ILC1 and ILC3 markers. During ischemia and myocarditis cardiac ILCs differentiated into ILC2s but not ILC1s and ILC3s. The number of ILC2s in the heart was associated with an increase of IL-33 production by cardiac fibroblasts. Furthermore, we found that non-cytotoxic cardiac ILCs are strictly cardiac resident cells during adulthood and circulating ILCs and ILCPs are unable to seed heart tissues.

## Results

### Innate Lymphoid Cells Type 2 Are Predominant in Heart of Patients With Ischemic Cardiomyopathy or Myocarditis

**Figure 1:**
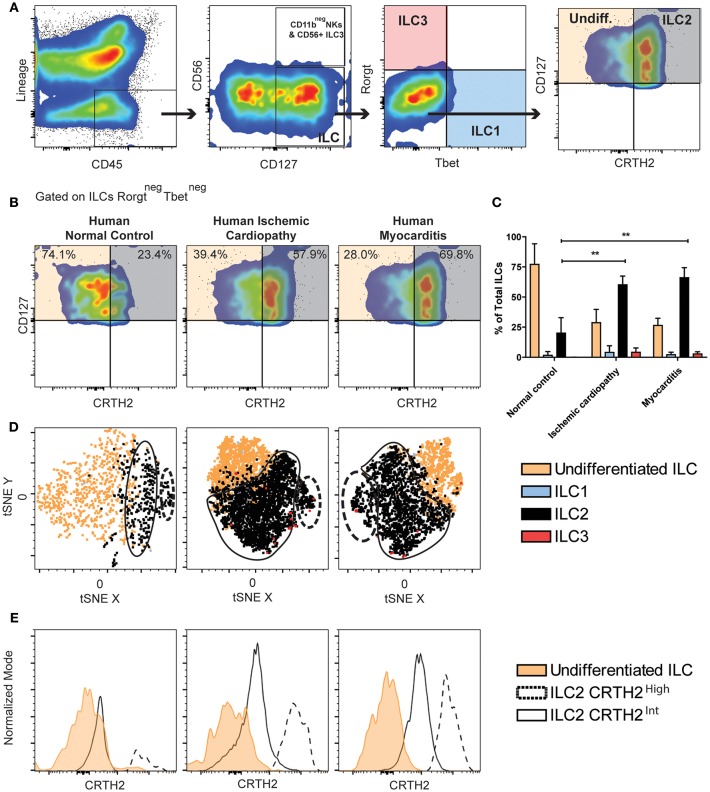
Majority of human ILCs are undifferentiated in healthy hearts, whereas ILC2s predominate in ischemic cardiomyopathy and myocarditis. **(A)** Human cardiac ILCs gating strategy, ILCs were defined as CD45^+^Lineage^neg^CD127^+^, NK cells as CD56^+^NKp44^+^, non-cytotoxic ILC1s (blue) as Tbet^+^, ILC2s (gray) as CRTH2^+^GATA3^int−high^, ILC3s (transparent red) as Rorγt^+^IL-23R^+^. Undifferentiated ILCs (transparent orange) were defined as ILCs negative for all type-specific markers. **(B)** Representative flow cytometry plots showing median examples of the profile of undifferentiated ILCs and ILC2s gated in Rorγt^neg^ Tbet^neg^ ILCs. **(C)** Frequency of ILC subsets in healthy human hearts, ischemic cardiomyopathy and myocarditis. **(D)** tSNE clustering analyses of ILCs immunophenotypes. **(E)** Comparison of CRTH2 expression in two distinctive ILC2s population in normal hearts, ischemic cardiomyopathy and myocarditis. Data show *n* = 5 for ischemic cardiomyopathy, *n* = 5 for myocarditis and *n* = 4 for controls. tSNE plots are concatenates of all the samples for group. Bar graphs shows Mean and SD. Statistics were calculated using Dunnett's test. **P < 0.01.

We used multiparameter flow cytometry to comprehensively characterize non-cytotoxic cardiac ILCs in human endomyocardial biopsy samples. Biopsies were taken from ischemic cardiomyopathy (*n* = 5) and myocarditis (*n* = 5) patients with heart failure during left ventricular assist device (LVAD) implantation. Both groups displayed similar clinical, hemodynamic, and echocardiographic features. The only significant difference between the groups was a lower mean age of the myocarditis patients (Table 1). Controls were rapid autopsy specimens from deceased patients without any cardiac pathology (*n* = 4). To exclude all lymphocytes, myeloid cells, other classic leukocytes subsets including CD11b^+^ NK cells, and other potential CD45^dim^ cells, we used a Lineage channel containing CD3, TCRαβ, CD20, CD11c, CD11b, CD123, BDCA2, CD14, FcεR1α, CD31, and CD34 in the flow cytometry gating strategy. Among CD45^+^Lineage^neg^ cells, non-cytotoxic ILCs were characterized as CD127 (IL-7R)^+^ (Figure 1A and Supplementary Figure 1). CD11b^+^ NK cells, a cytotoxic ILC1 subset, were excluded from this analysis with the Lineage cocktail, whereas CD11b^neg^ NK cells were excluded from the non-cytotoxic ILC analysis by gating on CD127^+^CD56^neg^NKp44^neg^ cells (59) (Figure 1 and Supplementary Figure 1). Non-cytotoxic ILC immunophenotypes were determined as ILC1 Tbet^+^, ILC2 CRTH2^+^, ILC3 Rorγt^+^IL23R^+^, or CD56^+^ (Figure 1A and Supplementary Figure 1) (59). The undifferentiated ILC population was CD127^+^CD45^+^Lineage^neg^ but also negative for all other cell type-specific markers such as CD56, NKp44, CRTH2, Rorγt, and IL23R (Figure 1A and Supplementary Figure 1). A significant proportion of non-cytotoxic ILCs was undifferentiated in normal controls, as well as in ischemic cardiomyopathy and myocarditis. The undifferentiated ILC population was predominant in normal controls, representing around 75% of the ILCs (Figures 1B,C). In ischemic cardiomyopathy and myocarditis, the undifferentiated ILC population decreased to about 25%, and ILC2 became the dominant population, representing around 65% of total ILCs (Figures 1B,C). That shift of undifferentiated ILCs to ILC2s was accompanied by minimal changes in non-cytotoxic ILC1 and ILC3 subpopulations (Figures 1B,C). In order to comprehensively characterize the entire ILC population and clearly dissect NK cells from the non-cytotoxic ILCs, we performed similar flow cytometry analyses in autopsy myocardial specimens, but placing CD11b in a separated channel out of the Lineage cocktail (Supplementary Figure 2). We confirmed that cardiac NK cells are mutually exclusive of non-cytotoxic ILCs, displaying a CD11b^+^CD56^+^NKp44^neg^CD127^neg^ profile. Conversely, non-cytotoxic ILCs were strictly CD127^+^. Following this alternative strategy, we found identical pattern of non-cytotoxic cardiac ILCs in the human heart. There were no ILC1 and ILC3 while 20% of cardiac ILC2s population were CRTH2^+^ ILC2s. The non-cytotoxic cardiac ILCs were mainly undifferentiated ILCs (~80%) (Supplementary Figure 2).

**Table 1:** Clinical and hemodynamic characteristics of patients.

**Variable**	**Ischemic cardiomyopathy**	**Myocarditis**	**Intergroup *P***
Number of patients	5	5	-
% of males	100%	100%	-
Age (years)	62.40 ± 2.73	35.00 ± 8.32	0.01
Height (cm)	173.60 ± 2.22	180.60 ± 3.04	0.10
Weight (Kg)	81.68 ± 5.41	96.42 ± 5.66	0.09
EF (%)	21.00 ± 2.44	16.00 ± 1.00	0.09
LVIDd (cm)	7.20 ± 0.21	7.25 ± 0.30	0.89
LVPWd (cm)	1.06 ± 0.02	0.87 ± 0.08	0.05
IVSd (cm)	0.94 ± 0.09	0.90 ± 0.08	0.76
BNP (pg/mL)	971.60 ± 531.10	1285.00 ± 536.70	0.71
Troponin level	5/5 < 0.15 pg/m	2/2 available < 0.15 pg/mL	-
Patients going to OHT	5/5	4/5 (1 patient to Jarvik)	-
Days on LVAD	339.80 ± 50.70	688.00 ± 208.10	0.14

*EF, Ejection fraction; LVIDd, Left ventricular internal diameter end diastole; LVPWd, Left ventricular posterior wall end diastole; IVSdL, Interventricular septum end diastole; BNP, Brain natriuretic peptide; OHT, Orthotopic heart transplant; LVAD, Left ventricular assist device*.

To better understand and validate the ILC2 and undifferentiated ILCs phenotype observed in the heart based on CRTH2 expression, we performed immunophenotyping of healthy human PBMCs (*n* = 3), (Supplementary Figure 3). We found a population of activated NKp44^+^CD127^neg^CD56^+^Tbet^+^ population distinct from non-cytotoxic circulating ILCs, which were mainly comprised of ILC2s (~60%) which homogenously expressed CRTH2. No undifferentiated ILC population was found in clear contrast to its dominant presence in the heart (Supplementary Figure 3).

Since cardiac ILCs are an extremely infrequent population, we performed analyzes of the immunophenotype clustering pattern of the non-cytotoxic cardiac ILC subpopulations using t-distributed Stochastic Neighbor Embedding (tSNE), a machine learning algorithm of dimensional reduction, in order to validate the conventional gating analyses. The clustering pattern in tSNE plots is considered a strong supportive evidence of the feasibility of those small subsets (60, 61). tSNE analyses were performed on concatenates of all the samples for each group, and a robust nucleated clustering of undifferentiated ILCs and ILC2s was observed (See Methods for technical details). ILC2 pattern in both ischemic cardiomyopathy and myocarditis showed two distinctive and significantly populated clusters (Figure 1D). The difference between those two clusters was the magnitude of CRTH2 expression (Figure 1E). We found an increase in the proportion of cells belonging to the cluster with a higher expression of CRTH2 in both diseases (Figures 1D,E). Thus, we observed increased proportion of the ILC2 population in the heart of patients with chronic ischemic cardiomyopathy and myocarditis, in contrast to a predominantly undifferentiated ILC profile in healthy human hearts.

### Cardiac ILC2 Population Increases in Murine Models of Myocardial Infarction and Myocarditis

**Figure 2:**
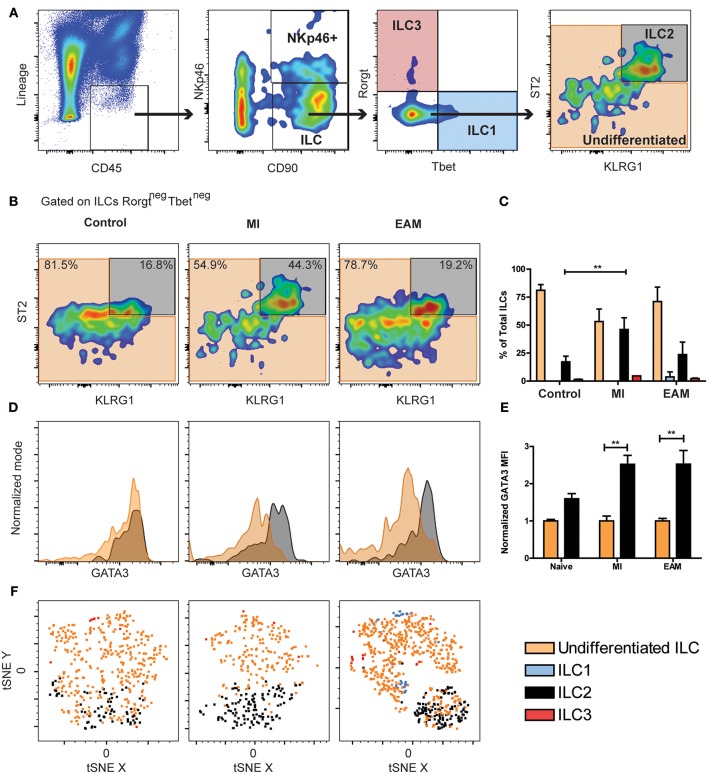
Undifferentiated ILCs are predominant in naïve mice hearts, while the ILC2 compartment expands during MI and EAM. **(A)** Mouse cardiac ILCs gating strategy, defining ILCs as CD45^+^Lineage^neg^CD90^+^, CD11b^neg^ NK cells and NKp46^+^ ILC3s as CD90^+^NKp46^+^, non-cytotoxic ILC1s (blue) as Tbet^+^, ILC2s (gray) as ST2^+^KLRG1^+^GATA3^int−high^, ILC3s (transparent red) as Rorγt^+^. Undifferentiated ILCs (transparent orange) as ILCs negative for all ILC type-specific markers. **(B)** Representative flow cytometry plots show median examples of the profile of undifferentiated ILCs and ILC2s in naïve, MI and EAM hearts gated on Rorγt^neg^ Tbet^neg^ ILCs. Frequency of ILC types in naïve, MI, EAM hearts. **(D)** Representative flow cytometry histograms show GATA3 expression on ILC2s (black) and undifferentiated ILCs (orange). **(E)** Normalized GATA3 MFI in naïve, MI, and EAM hearts. **(F)** tSNE analysis shows ILC2 clustering (black) within the undifferentiated population (orange). Plots, including tSNE analyses, show concatenates of all mice in each group of 1 of 3 independent experiments (*n* = 4–5 group / experiment). Bar graphs shows Mean and SD. Statistics for **(C,E)** calculated by Dunnett's test. **P < 0.01.

Next, we examined murine cardiac ILCs in two models of cardiac diseases, myocardial infarction (MI) and experimental autoimmune myocarditis (EAM), using the flow cytometry approach. Severity of EAM at day 21 was assessed with standard histology (H&E, Supplementary Figure 4A). The gating strategy followed the same rationale as human experiments, while using the standardized markers for mouse ILCs. Mouse ILCs were defined as CD45^+^Lineage^neg^CD90^+^, where the Lineage was CD3, TCRβ, CD5, CD19, CD11b, CD11c, GR1, FcεR1α, CD31, and TER119, to exclude other CD45^+^ populations, erythroid and endothelial cells. The CD11b^+^ NK population was excluded by the presence of CD11b in the Lineage cocktail. Downstream in the gating strategy, CD11b^neg^ NK cells were identified by NKp46 positivity and further Rorγt evaluation. Within the non-cytotoxic ILCs (CD45^+^Lineage^neg^CD90^+^NKp46^neg^) the classic subpopulations were characterized as Tbet^+^ ILC1s, ST2^+^KLRG1^+^GATA3^int−high^ ILC2s, Rorγt^+^ ILC3s and Tbet^neg^Rorγt^neg^ST2^low^KLRG1^neg−int^ undifferentiated ILCs population (Figure 2A). We did not find differences in the composition of the cardiac ILCs compartment between naïve, mock immunized and sham surgery controls (Supplementary Figure 4B). Within the CD90^+^NKp46^+^ population we did not found Rorγt, which excluded the existence of NKp46^+^ ILC3s (Supplementary Figure 4C). The ILC population did not express Rorγt or IL25R nor hyper-high levels of KLRG1 (Figure 2A and Supplementary Figure 4D). Thus, we ruled out an inflammatory ILC (i-ILC2) and ILC3 identity (Figures 2A,B and Supplementary Figure 4D). Unlike peripheral ILCPs (47), the cardiac ILC population was CD117 (cKit) negative (Supplementary Figure 4D). Around 75% of cardiac ILCs were undifferentiated in naïve controls, resembling the findings in human endomyocardial biopsy samples (Figures 1B, 2B). We found a significant increase of ILC2 to about 45% of total cardiac ILC population in MI, paralleled by a decrease in the undifferentiated ILC population (Figures 2B–C). The proportion of ILC1s and ILC3s was negligible. In EAM, although the flow cytometry plot showed a qualitatively more robust ILC2 differentiation with stronger ST2 expression, the increase in the proportion of this population was not statistically significant relative to controls (Figures 2B–C). Nevertheless, the absolute ILC count in the heart demonstrated a significant increase in both, undifferentiated and ILC2s in EAM as compared to controls (Supplementary Figures 4C,D). In fact, the increase in the absolute number of cardiac ILC2 in MI was comparable to that in EAM (Supplementary Figure 4E). In EAM, the undifferentiated population also expanded significantly (Supplementary Figure 4F). ILC2s had a higher GATA3 expression as compared with undifferentiated ILCs in all conditions, although undifferentiated ILCs intermediately express GATA3 (Figure 2D). GATA3 mean fluorescent intensity (MFI) expression in EAM and MI was normalized to the mean GATA3 MFI of naïve controls. The normalized GATA3 expression by ILC2s in naïve hearts was 1.5 higher than the mean of undifferentiated ILCs (Figure 2E). In MI and EAM hearts, we observed a 2.5-fold upregulation of GATA3 in the expanded ILC2 population, compared to undifferentiated ILCs (Figure 2E). To confirm the reliability of such a small population, we validated the conventional gating strategy with tSNE analyses of concatenated samples for each experimental group, following the same approach used with human samples. We also found a robust clustering of the undifferentiated and ILC2 population, thus supporting the homogeneity of each subset and ruling out significant noise interference (Figure 2F).

In order to precisely discriminate NK cells from non-cytotoxic ILCs, we analyzed non-cytotoxic ILCs and NK cells in the hearts of WT Balb/c mice, placing CD11b and CD3 out of the Lineage cocktail (Supplementary Figure 5A). We found that the vast majority of NK cells were CD11b^+^CD90^neg^CD122^+^. Importantly, the CD11b^neg^CD90^+^NKp46^+^ population was strictly an NK population and not an ILC3 subset, as it displayed a downstream CD122^+^Rorγt^neg^ status, thus confirming again the absence of ILC3 in murine heart tissues (Supplementary Figure 5A). With this alternative gating strategy, we found an identical pattern amongst non-cytotoxic ILCs as in the previous experiments, with predominance of undifferentiated ILCs over ILC2s (Supplementary Figure 5B).

Despite we observed a significantly more robust expansion of ILC2s percentagewise during myocarditis in humans as compared with murine EAM, the predominance of undifferentiated ILCs and the overall composition of this compartment in naïve mouse hearts resembled the human controls. Also, a similar expansion of differentiated ILC2s was observed in murine and human ischemic heart diseases. Thus, our findings suggest that heart ILCs are a quiescent and phenotypically undifferentiated population which develop ILC type 2 features during inflammatory processes such as ischemia and autoimmunity.

### A Subset of Cardiac Fibroblasts Express IL-33 During MI and EAM

**Figure 3:**
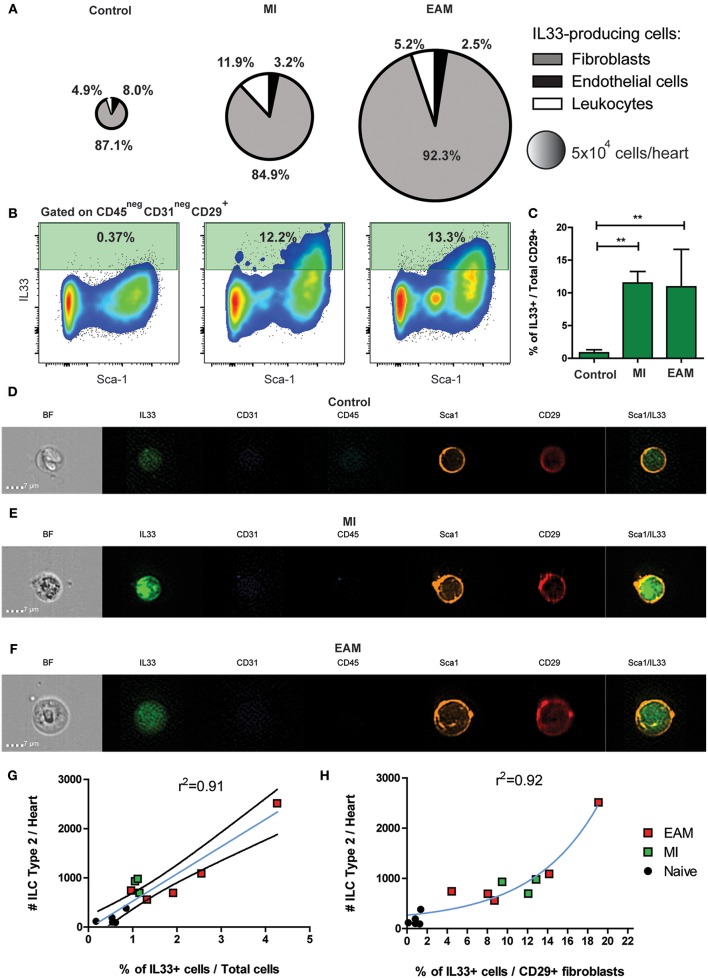
IL-33 is expressed by cardiac fibroblasts during MI and EAM. **(A)** Pie charts represent the percentage and total number of the heart IL-33-producing population and its composition in naïve, MI, and EAM hearts. Charts are based on concatenates of all mice in a group. **(B)** Representative flow plots of IL-33 expression by CD45^neg^CD31^neg^CD29^+^ fibroblasts are shown for all conditions. Median examples are shown. **(C)** Frequency of IL-33 producing cardiac fibroblasts in naïve, MI and EAM hearts. **(D–F)** Representative examples of Microscopy flow cytometry (ImageStream) single cells images, showing the pattern of IL-33, Sca-1, and CD29 expression in cardiac fibroblasts. Representative examples of 500–1,500 events per sample are shown. **(G)** Linear regression of absolute number of ILC2s and percentage of IL-33^+^ cells in naïve, MI, and EAM hearts analyzed together. Blue line shows linear function and black lines the 95% confidence interval. **(H)** Regression plot shows an exponential correlation between absolute number of ILC2s and percentage of IL-33^+^ cells amongst fibroblasts, represented by the blue line. Data of 1 of 2 independent experiments, *n* = 5 for controls and EAM, *n* = 3 for MI. Bar graphs shows Mean and SD. Correlations estimated using Pearson r. Statistic difference of means were calculated with Dunnett's test. **P < 0.01.

IL-33 is the main stimulus for ILC2 differentiation and expansion (33, 62). We found that in both humans and mice, ILC2 expanded during cardiac ischemia and myocarditis despite the differences in the initial insult. We used a knock-in IL-33 reporter mouse strain (*il33*^*citrine*/+^) to determine the source of IL-33 during MI and EAM. A small proportion of cardiac resident cells constitutively express IL-33 in naïve state (Figure 3A). The number of IL-33-producing cardiac cells significantly expanded during MI and EAM, about 6-fold and 10-fold, respectively (Figure 3A). In all conditions, the predominant IL-33^+^ cells were CD45^neg^CD31^neg^CD29^+^ cardiac fibroblasts (Figure 3A). Within the cardiac fibroblasts, the IL-33 production was restricted to the Sca-1^+^ subset (Figure 3B). The proportion of IL-33^+^ cells among fibroblasts showed a 20-fold increase in MI and EAM compared to controls (Figure 3C). tSNE analysis demonstrates a well-defined cluster of Sca-1^+^ cardiac fibroblasts, representing about 50% of total CD29^+^ fibroblasts (Supplementary Figure 6). The sub-population of IL-33^+^ fibroblasts was entirely restricted to the cluster of Sca-1^+^ fibroblasts (Supplementary Figure 6). We used microscopy flow cytometry (ImageStream) to verify the reliability of the signal of the IL-33 reporter molecule (citrine) and visually corroborate the conventional flow cytometry results. We confirmed the predominance of Sca-1^+^ fibroblasts as the source of IL-33. The IL-33/citrine signal showed the expected homogeneous intracellular pattern, thus ruling out auto-fluorescence phenomena. Even within the IL-33^+^ cells, the citrine intensity was dimmer in controls as compared with MI and EAM (Figures 3D–F). The absolute number of ILC2s in the heart showed a linear correlation with the proportion of IL-33 producing cells amongst total cellularity in all conditions (Figure 3G), whereas the ILC2s' absolute count had an exponential correlation with the proportion of IL-33^+^ cells within the fibroblasts (Figure 3H). These results strongly suggest that, regardless of the type of noxa (autoimmune or ischemic), IL-33 is produced by a subset of cardiac fibroblasts during tissue damage, leading to a differentiation of quiescent cardiac ILCs and expansion of the ILC2 compartment.

### Naïve Cardiac ILCs Are a Type-2 Committed Population With Restricted Plasticity

**Figure 4:**
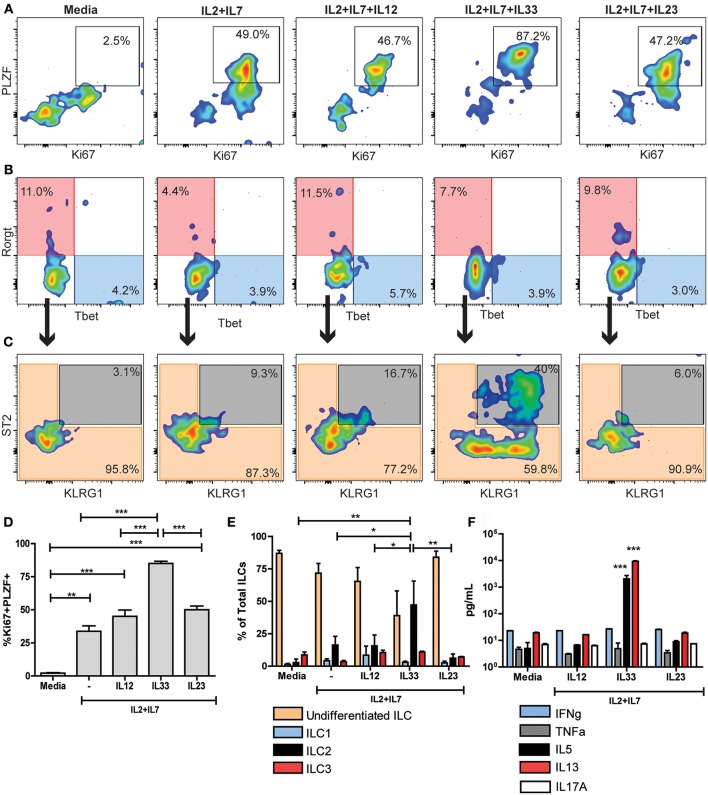
Naïve cardiac ILCs are an ILC type-2 committed population. **(A)** PLZF/Ki67 activation pattern of ILCs under different *in vitro* conditions: Media, IL-2+IL-7, IL-2+IL-7+IL-12 (to induce ILC1s), IL-2+IL-7+IL-33 (to induce ILC2s) and IL-2+IL-7+IL-23 (to induce ILC3s). **(B)** Flow cytometry plots show ILC1 (blue) and ILC3 (transparent red) differentiation under different conditions, based on Rorγt and Tbet expression. **(C)** Plots show undifferentiated ILCs (transparent orange) and ILC2s (gray) compartments after *in vitro* stimulation, based on KLRG1 and ST2 expression. **(D)** Comparison of PLZF^+^Ki67^+^ activated population in different conditions. **(E)** Comparison of different ILC subpopulations after *in vitro* stimulation. **(F)** Comparison of cytokine profile determined by ELISA under different stimulating conditions. In *F*, ***indicates significance of both bars with respect to all other conditions. Flow cytometry plots show median representative examples. Graphics show results of 1 of 4 independent experiments, each one made in triplicates for each condition. Bar graphs shows Mean and SD. Statistics calculated with one-way ANOVA and Bonferroni *post-test*. *P < 0.05; **P < 0.01; ***P < 0.001.

To evaluate the functional capacity and plasticity of the predominantly undifferentiated non-cytotoxic ILC population in a naïve mouse heart, we performed *in vitro* differentiation experiments. We FACS sorted CD45^+^Lineage^neg^CD90^+^NKp46^neg^ ILCs from naïve mice hearts. Then, ILCs were stimulated with different cytokines for 6 days to induce type-specific differentiation. To induce ILC1 differentiation, we used recombinant cytokines IL-2+IL-7+IL-12, to generate ILC2s we used IL-2+IL-7+IL-33 and for ILC3 induction, IL-2+IL-7+IL-23. As controls we included plain media and IL-2+IL-7 only. ILCs in media only were undifferentiated with mild expression of Ki67 and no expression of the PLZF (Figure 4A). Unspecific stimulation with IL-2+IL-7 induced a moderate activation of cardiac ILCs, characterized by co-expression of Ki67 and PLZF (Figure 4A), but no significant Rorγt, Tbet, or ST2 and KLRG1 expression (Figures 4B,C). The proportion of ILC2s under stimulation with IL-2+IL-7 was slightly higher than plain media (Figure 4C). ILCs stimulated with type 1, 2, and 3 differentiating conditions were activated (Figure 4A), reaching statistical significance in every case in respect to the media control (Figure 4D). Nevertheless, the proportion of activated PLZF^+^Ki67^+^ cells was significantly higher with the IL-33 stimulation as compared to controls as well as IL-12 and IL-23 conditions (Figure 4D). Unexpectedly, IL-12 and IL-23 were unable to induce ILC1 and ILC3 differentiation, respectively (Figure 4B). No differences were observed between controls, stimulation with IL-23 and IL-1β (Supplementary Figures 7A–E). IL-33, in conjunction to IL-2+IL-7, induced a robust ILC2 differentiation (Figures 4A,C,E). Finally, as a functional readout, the main ILC1, 2, and 3 cytokines were analyzed in the supernatants by ELISA. Congruent with the immunophenotype, neither IFNγ, TNFα nor IL-17A increased in any of the conditions and remain at basal levels (Figure 4F and Supplementary Figure 7F). IL-33 induced a marked and significant production of IL-5 and IL-13 (Figure 4F).

The monotonic PLZF/Ki67 co-expression (~85%) achieved only in IL-33 conditions suggests that the entire non-cytotoxic cardiac ILC population, including the undifferentiated ones, have a type 2-biased functionality evidenced by responsiveness to IL33. To rule out the possibility of exclusive expansion of already differentiated ILC2 during this experiments, we FACS sorted CD45^+^Lineage^neg^CD90^+^NKp46^neg^ST2^+^ cardiac ILCs and culture them with IL-2+IL-7+IL-33 for 6 days. That small subset failed to significantly proliferate and differentiate (Supplementary Figure 7G), thus strongly suggesting that undifferentiated ILC population, or at least a subset of them, can differentiate to a full ILC2 status, but not to ILC1 or ILC3 phenotype.

These results confirm that naïve non-cytotoxic cardiac ILC population is committed with an ILC2 fate and functionality, despite its undifferentiated status in steady-state conditions.

### Naïve Cardiac ILCs Transiently Express PLZF Upon Activation and ILC2 Differentiation

**Figure 5:**
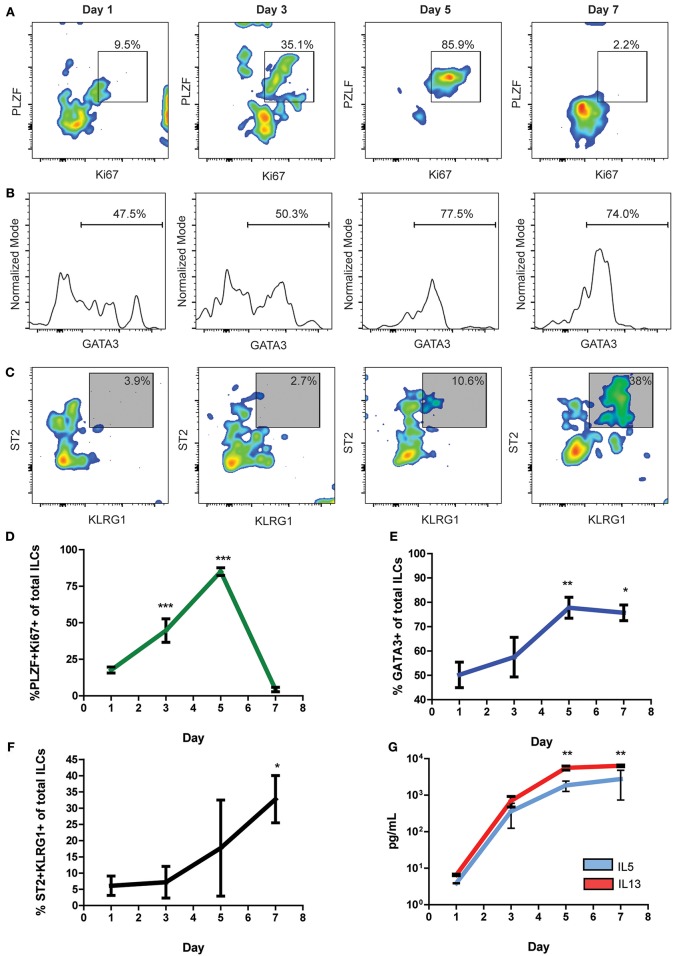
Undifferentiated naïve cardiac ILCs transiently express PLZF upon activation. **(A)** Timeline of PLZF^+^Ki67^+^ activation pattern under *in vitro* ILC2-differentiating conditions, IL-2+IL-7+IL-33. **(B)** Progression of GATA3 expression, showing the gate of GATA3^+^ ILCs. **(C)** Time course of ILC2 phenotype development, based on KLRG1 and ST2 co-expression. **(D)** Graph representing the evolution of the PLZF^+^Ki67^+^ co-expression. **(E)** Timeline of GATA3 expression during *in vitro* IL-33 stimulation. **(F)** Time curve showing the progression of ILC2 phenotype development. **(G)** Curve showing the evolution of IL-5 (blue) and IL-13 (red) production under IL-2+IL-7+IL-33 stimulation, as measured by ELISA of supernatants. Graphics show results of 1 of 2 independent experiments, each one made in triplicates for each condition. Asterisks represent the statistical significance of each time-point with respect to day 1-baseline values. Dots and whiskers show Mean and SD. Statistics calculated with one-way ANOVA and Bonferroni *post-test*. *P < 0.05; **P < 0.01; ***P < 0.001.

PLZF has been described as a nuclear factor transiently expressed by subsets of ILC progenitors during activation, including the subset of ILC2-commited progenitors (42). As naïve cardiac ILCs were predominantly undifferentiated but ILC2-commited, we decided to perform timeline *in vitro* experiments to study the dynamics of their activation and steady-state properties. We determined the kinetics of Ki67 and PLZF expression to address the naïve cardiac ILCs' proliferation capacity and progenitor features (Figure 5A). Also, we analyzed the progression of the ILC2-associated transcription factor GATA3 (Figure 5B) and the evolution of ILC2 phenotype (Figure 5C) over time under the influence of IL-33. Naïve cardiac ILCs CD45^+^Lineage^neg^CD90^+^NKp46^neg^ were FACS sorted and cultured *in vitro* with IL-2+IL-7+IL-33, and its phenotypic and functional changes assessed on days 1, 3, 5, and 7. The activation dynamics showed a low baseline ILCs activation at day 1. Ki67^+^PLZF^+^ co-expression progressively increased, reaching significance at day 3 and peaking at day 5 (Figures 5A,D), when a monotonic co-expression was achieved. PLZF and Ki67 levels abruptly decayed after day 5, returning to baseline levels by day 7, demonstrating a pattern of transient PLZF expression after cytokine-induced activation (Figures 5A,D). GATA3, however, showed different kinetics than PLZF, as it progressively increased, reaching a plateau at day 5 and persisting significantly elevated at day 7 (Figures 5B,E). The ILC2 phenotype developed progressively in terms of KLRG1 and ST2 co-expression, following a quasi-linear dynamic after day 3 (Figures 5C,F). Finally, the production of IL-5 and IL-13 increased in parallel following an exponential pattern (Figure 5G). Therefore, we found that cardiac ILCs, in addition to having an undifferentiated phenotype and negligible cytokine production in resting conditions, display a progenitor-like activation pattern, implying transient expression of PLZF. This behavior suggests an incomplete, yet biased, differentiation in normal conditions, compatible with a committed progenitor status.

### Cardiac ILCs Are a Strictly Resident Population

**Figure 6:**
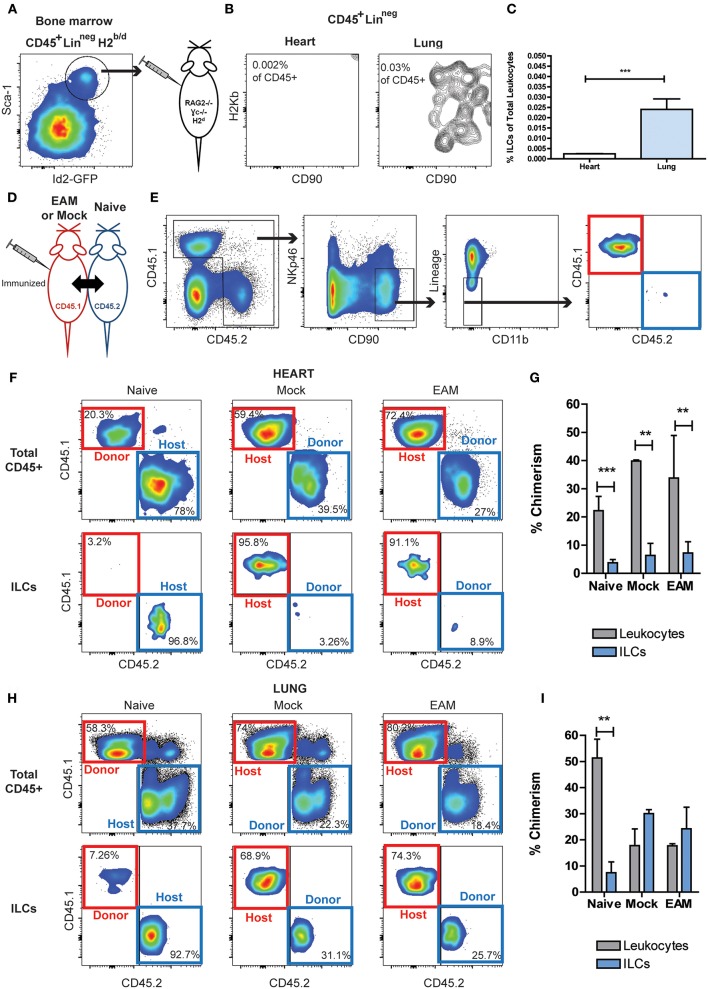
Circulating ILCs cannot infiltrate heart tissues. **(A)** FACS sorting strategy of bone marrow ILC progenitors, CD45^+^Lineage^neg^Id2^+^Sca1^+^, transferred into RAG2^−/−^γc^−/−^. **(B)** Flow cytometry plots of infiltrating H2K^b^ ILCs in heart and lung. **(C)** Bar graphs comparing the detectable infiltrating ILC population in heart and lung. **(D)** Scheme of parabiosis experiment. **(E)** Gating strategy used to analyze ILC population at the peak of EAM, using a naïve parabiont as representative example. In naïve pairs the infiltrating ILCs were CD45.1 (red boxes), and in Mock and EAM pairs the infiltrating ILCs were CD45.2 (blue boxes). **(F)** Flow cytometry plots showing the source of leukocytes and ILCs in heart. **(G)** Bar graphs comparing the proportion of infiltrating ILCs and leukocytes in heart. **(H)** Flow cytometry plots showing the source of leukocytes and ILCs in lung. **(I)** Bar graphs comparing the proportion of infiltrating ILCs and leukocytes in lung tissues. Naïve *n* = 8, EAM *n* = 3, Mock *n* = 2. Bar graphs shows Mean and SD. Statistics were calculated with Student t **(C)** and one-way ANOVA and Bonferroni *post-test* **(G–I)**. **P < 0.01, ***P < 0.001.

To differentiate resident vs. infiltrating status of cardiac non-cytotoxic ILCs, we performed adoptive transfer experiments. Bone marrow ILC progenitors, Lineage^neg^CD45^+^Id2^+^Sca-1^+^ cells were FACS sorted from semi-allogeneic H2^b/d^ Id2^GFP^ reporter mice and transferred into RAG2^−/−^ γc^−/−^ H2^d^ mice, allowing to track the transferred ILCs based on H2-K^b^ expression (Figure 6A). After 28 days of the transference, no heart-infiltrating ILCs were detected, but we found a small but well-defined infiltrating ILC population in lung tissues (Figures 6B,C). To further investigate the origin of cardiac ILCs, we performed parabiosis experiments. In this experimental setting, CD45.1 mice underwent EAM induction (on days 0 and 7), and were surgically paired to CD45.2 naïve mice on day 2. Pairs with a mock immunized CD45.1 mouse and naïve CD45.1 were used as controls (Figure 6D). In naïve parabionts, the chimeric cells were CD45.1, whereas in mock and EAM parabionts, the chimeric cells were tracked based on CD45.2 expression (Figure 6E). We checked establishment of leukocyte chimerism in peripheral blood at day 14 in every case, finding a successful engraftment of 30–40% (not shown). On day 21, all groups had leukocyte chimerism in their hearts, about 20% in naïve parabionts and 35–40% in mock and EAM parabionts (Figures 6F,G). Nevertheless, the chimerism in the cardiac ILC compartment was disproportionately low as compared with the total leukocyte engraftment. In all conditions, the chimeric ILCs represented about 5% of the heart ILC population (Figures 6F,G). Leukocyte mixed chimerism also occurred in lungs, with 50% engraftment in naïve parabionts and about 20% in mock and EAM parabionts. In naïve parabionts, the specific pulmonary ILC engraftment was also disproportionally low as compared with the leukocyte chimerism, representing about 8% of the ILC population compared to 50% of leukocyte population. Conversely, in mock and EAM mice, both known to have systemic inflammation due to use of CFA as adjuvant (63), the ILC engraftment in the lungs was proportional and not statistically different as compared to the whole leukocyte chimerism, representing both 20–30% of the ILC and leukocyte compartments, respectively (Figures 6H,I). Lung engraftment demonstrated the intrinsic infiltrative capacity of circulating ILCs. These adoptive transfer and parabiosis results suggest that heart is a unique niche with resident ILCs that are not replenished from blood ILCs even during cardiac inflammation during myocarditis or after ischemia.

## Discussion

ILCs are a subset of leukocytes which play a key role in multiple immune processes, providing a link between the innate and the adaptive responses (2). Originally, ILCs were considered patrollers of mucosa- and epithelium-associated tissues, such as the respiratory tract, gut, and skin (15). Further investigation has revealed their presence and physiologic importance in organs, such as the liver and adipose tissue, having an impact even in modulating metabolic processes (20, 21, 23). In this paper, we investigated characteristics of heart ILCs.

We found that in normal human heart and naïve mouse tissues, cardiac ILCs lack a well-defined immunophenotype, and we denoted them as undifferentiated. Those ILCs did not express ILC1 nor ILC3 markers. In normal human hearts, the majority of cardiac ILCs were undifferentiated as they did not express the ILC2-specific marker CRTH2 (64). In naïve mice, the predominant undifferentiated cardiac ILCs expressed intermediate levels of GATA3 and variable levels of KLRG1. They did not express ST2 as conventional ILC2s do. Undifferentiated ILCs neither expressed high levels of KLRG1, IL25R (IL-17RB), nor Rorγt as is characteristic for inflammatory ILC2s (i-ILC2s) (34). The predominance of ILCs without a fully mature phenotype in normal hearts is different from the typical complete ILC differentiation described in organs such as the skin, lung, and gut (65). Our findings support the concept that normal ILCs status depends on an organ-specific milieu leading to a tissue-specific ILC training, a phenomenon previously described as “ILC-poiesis” (47).

Inflammation associated with ischemic cardiomyopathy is considered to be modulated by a monocyte/macrophage response and a cytotoxic, Th1, Th17, and Treg responses (54, 55, 66–70). Autoimmune myocarditis predominantly exhibits Th1 and Th17 responses during the inflammatory phase, with Th17 activity being required for the progression to dilated cardiomyopathy (DCM) in chronic stages (58, 71, 72).

We found a robust increase of classic ILC2s, which replaced a majority of the undifferentiated ILC population in a group of patients with both ischemic- and myocarditis- end-stage heart failure. Remarkably, almost no ILC1s and ILC3s were detected, despite the differences in the etiology of both diseases and the fact that both Th17 and Th1 responses are essential for their pathogenesis. We found significant similarities in the non-cytotoxic ILC compartment between human and murine hearts in steady-state and ischemic conditions. Regarding myocarditis, the expansion of ILC2s was significantly more robust in human cardiomyopathy than in murine EAM, which might be an evidence for inter-species differences. This finding could be also influenced, by the chronicity of the analyzed human myocarditis as compared with the EAM model.

ILCs are influenced by the complex interaction of microbiota, external environment, stromal, parenchymal, and immune cells (9, 73, 74). Our experimental data strongly suggest that cardiac ILCs are biased toward an ILC2 fate, regardless of the differences in the pathologic T cell milieu and despite the undifferentiated ILC status in steady-state physiologic conditions. Furthermore, IL-33 reporter mice showed a massive increase in IL-33 production by Sca-1^+^ cardiac fibroblasts during ischemia and myocarditis, which was correlated with an expansion of the ILC2 compartment. These findings complement our previous reports about the immunologic importance of Sca-1^+^ cardiac fibroblasts and its role in inflammatory heart diseases, such as the influence of Sca-1^+^ cardiac fibroblasts in the development of heart failure via secretion of GM-CSF (55).

We propose that, regardless of the triggers and initial noxa, cardiac fibroblasts produce IL-33 as an alarmin, inducing the final differentiation of the ILC2-biased population. Thus, in the heart, the ILC2 development is not restricted to Th2-predominant inflammatory processes. This ILC cardiac-specific feature might lead to diversification and deviation of the immune response, and contribute with an innate component in acute and chronic inflammatory heart diseases.

In addition, we found the existence of a baseline low levels of IL-33 production in heart tissues in normal physiologic conditions. We hypothesize that this is one of the components of the heart micro-environment that generates the ILC2-bias of the quiescent undifferentiated cardiac ILCs. Similar phenomenon seems to occur in the lung, but in this case the exposure to external environment leads to complete ILC2 differentiation (74).

*In vitro* experiments demonstrated that activation of undifferentiated cardiac ILCs was characterized by a transient co-expression of Ki67 and PLZF, as described for ILC progenitors (42, 43). Nevertheless, cardiac ILCs did not express CD117 (cKit) nor have multipotent capacity, unlike the peripheral ILCPs present in cord blood, lung, and liver (47). Despite the fact that cardiac ILCs expressed PLZF under the influence of only IL-2 and IL-7, the presence of IL-33 in the milieu was strictly required for final ILC2 development. As a consequence, the phenotype and functionality of cardiac ILCs are not compatible with previously described peripheral ILCPs. Conversely, cardiac ILCs are a non-multipotent population that retain restricted progenitor-like features, such as the capacity to express PLZF and lack of completely differentiated phenotype in normal conditions.

The residence and infiltration properties of ILCs are still a matter of debate. The preferential role of tissue resident ILCs in early stages of the inflammatory responses is widely accepted (15). Nevertheless, their migratory and infiltrative capacities have also been described (47, 65). We showed here, using adoptive transfer experiments of bone marrow derived-ILC progenitors, that ILCs have an intrinsic infiltrative capacity in adult lymphoid-deficient mice RAG2^−/−^ γc^−/−^, as was evident in lung tissues. Conversely, bone-marrow-derived ILC progenitors were unable to seed the heart. Similarly, we demonstrated that circulating chimeric ILCs were able to populate lungs in naïve, mock and EAM parabionts, but were unable to seed heart tissues. These findings suggest that heart is a restricted niche for ILCs, and the limited traffic is a heart-specific phenomenon rather than an intrinsic feature of migratory ILCs.

Overall, our study shows that the heart is a unique niche in terms of the ILC compartment. Cardiac ILCs seem to be strictly resident population in adulthood. It remains to be elucidated in which stage of fetal development or early life the heart is populated by ILCs, a complicated conundrum due to the complexity of the embryologic development of the heart (52). Cardiac ILC resident population is constituted by quiescent ILC2-committed undifferentiated cells, which remain in resting status in physiologic conditions. Importantly, heart inflammation of multiple etiologies and Th skewness, such as ischemia and autoimmunity, activates a fibroblast-IL-33-ILC axis, which induces activation and differentiation of ILC2s.

IL-33 production in heart has been shown in other pathologic conditions, such as pressure overload (30). Soluble ST2, thought to be a homeostatic neutralizing molecule, has been described as a biomarker of heart failure of any etiology (75, 76). These facts prompt us to propose the existence of a heart stroma/parenchima-IL-33-resident ILC axis that deserves to be explored as a biomarker and potential therapeutic target for multiple inflammatory heart diseases. Our data show that important features, such as the balance between tissue resident and infiltrating ILCs, their status in physiologic conditions, and their final fate do not depend only on intrinsic ILC's cellular capacities, but also on organ-specific properties and microenvironments. In this regard, our findings are aligned with recent theoretical proposals about the dynamic changes of the innate lymphoid compartment over time and in tight association with organ-specific and microenvironmental features (77), which might have implications in physiologic homeostasis and pathologic processes.

## Materials and Methods

### Human Samples

#### Heart Samples

Endomyocardial biopsies from the apex of the left ventricle were obtained from patients with end stage heart failure (American Heart Association, AHA, stage D) due to chronic ischemic cardiomyopathy or secondary to progression of myocarditis-related cardiomyopathy undergoing implantation of LVAD devices at the Texas Heart Institute. Informed consent was obtained from human subjects and the study protocol was approved by the Committee for the Protection of Human Subjects (University of Texas Health Science Center at Houston. IRB #HSC-MS-05-0074) as previously described (78). Samples were properly preserved in cryovials embedded in liquid nitrogen (−190°C) and then kept at −80°C in the tissue bank at the Texas Heart Institute, Houston, TX. Aliquots of the samples were shipped frozen and processed for flow cytometry in Dr. Ciháková lab, at Johns Hopkins University, Department of Pathology, Baltimore, MD. Reported diagnoses correspond to clinical charts, based on standard histology (H&E), clinical presentation, hemodynamic parameters, and routine clinical biochemical and serology parameters. Patient's information of all samples was processed with random non-linked code relabeling in a database at the time of preservation. Furthermore, for each sample, a separate random non-linked code was assigned for the analyses process. Autopsy samples were obtained upon request, with a time of ischemia of 16–24 h from the Department of Pathology, Johns Hopkins Hospital, aliquot and frozen in cryovials at −80°C.

#### Blood Samples

Approximately eight milliliters of blood was drawn under aseptic conditions from consenting healthy volunteers from the basilic vein in the cubital fossae, and collected in CPT™ cell tubes (BD Vacutainer, Ref# 362753). Then, tubes were spin at room temperature (~21°C) at 1,800 g for 30 min without deceleration. Then the layer of PBMCs was gently removed, transferred into conical tubes and washed in PBS. After adjusting cell concentration, the PBMCs were processed in fresh for immunostaining.

### Mice

All purchased mice were obtained at 6–10 weeks old, and all mice used for experiments were 8–10 weeks old for EAM, mock and naïve conditions and 9–11 weeks old for MI and sham surgery. We purchased the following strains from Jackson labs: WT Balb/cJ mice (JAX000651), CD45.1 WT Balb/cJ mice (JAX006584), RAG2^−/−^γc^−/−^ mice on Balb/c background (JAX014593), Id2 reporter mouse on C57BL/6 background (B6.129S(Cg)-Id2tm2.1Blh/ZhuJ, JAX016224). Those C57BL/6 Id2 reporters were crossed with CD45.1 Balb/c (JAX006584) to obtain semi-allogeneic H2^b/d^ CD45.1^+^ Id2^GFP/+^ reporter mice in F1. Genotyping of Id2 alleles was done by PCR following vendor instructions (primers: common forward CAAGAAGGTGACCAAGATGGA; common TCTGGGCAGTGGCGTACTT; forward mutant GATCACTCTCGGCATGGACG), and H2 haplotyping and CD45.1 cogenic expression by FACS. *il-33*^*citrine*/*citrine*^ mice on Balb/c background (*Il33*^*tm*1*Anjm*^) (79) were obtained from Dr. Andrew McKenzie, MRC Center Cambridge). To obtain IL-33 citrine reporter animals (*heterozygous il-33*^*citrine*/+^), we crossed Balb/c *il-33*^*citrine*/*citrine*^ mice with WT Balb/cJ mice; whereas functionally IL-33 KO mice were obtained by following a homozygous x homozygous crossing scheme (*il-33*^*citrine*/*citrine*^ × *il-33*^*citrine*/*citrine*^). For terminal experiments, mice were euthanized by cervical dislocation after achievement of deep anesthesia status with a single intra-peritoneal dose of Avertin (Tribromoethanol 2.5% w/v; dose of 0.02 mL/gram of body weight).

### EAM Induction

To induce EAM, we injected mice with 125 μg of α-myosin heavy chain peptide (MyHCα614-629, Ac-SLKLMATLFSTYASAD) emulsified in CFA supplemented with 4 mg/mL heat-killed *Mycobacterium tuberculosis* strain H37Ra on days 0 and 7. On day 0, mice also received a dose of 500 ng pertussis toxins intraperitoneally (57, 71, 80).

### EAM Histology Assessments

Myocarditis severity was evaluated by histology on day 21. Heart tissues were fixed in SafeFix solution, paraffin embedded and then cut in 5 μm serial sections. Sections were stained with H&E and ventricular inflammation was evaluated with light microscopy, and scores from two independent evaluators (DC and WBB) were averaged using the following criteria: grade 0, no inflammation; grade 1, <10% of the heart section is involved; grade 2, 10–25%; grade 3, 25–50%; grade 4, 50–75%; grade 5, >75% (57).

### Myocardial Infarction

To induce MI, we performed permanent ligation of the left anterior descending coronary artery or to a sham operation without ligation. Briefly, mice were anesthetized with 3.5% isoflurane, endotracheal intubation performed and then mechanical ventilation started and kept throughout the operation via small animal ventilator (Harvard Apparatus, model 845). Pre-operational analgesics (0.05 mg/kg Buprenorphine) and paralytics (1 mg/kg Succinylcholine) were administrated prior to operation. A thoracotomy was performed on the 3rd or 4th intercostal space. A 8-0-polyethylene suture was advanced sub-epicardially and perpendicular to the left anterior descending coronary artery. For permanent occlusion, a ligation was done around the artery. The immediate impact was verified by myocardial bleaching and decreased contractility below the occlusion. The chest and skin were closed with a 6-0 nylon suture. Those procedures were performed by the same surgeon, who was also blinded to the experimental design (GC). Mice that died during recovery from anesthesia were excluded from the analysis. Sham-operated animals underwent a similar procedure but without coronary artery ligation (55).

### Flow Cytometry (FACS), Imaging Flow Cytometry (ImageStream), and FACS Sorting

Human myocardial biopsy samples weight ranged between 80 and 300 mg. Mouse heart and lungs were perfused *in situ* with PBS using a peristaltic pump (Rainin PR-1), via left and right ventricles for 5–6 min or until the organs looked bleached and pale. In murine experiments approximately half of mice hearts (sagittal cut) were used for FACS, having a weight between 70 and 90 mg; whereas the remaining cardiac tissues were used for histology. Heart and lung FACS specimens were cut in fragments of ~2 mm^3^. Fragments were placed in GentleMACS C Tubes (Miltenyi Biotec) in 5 mL of enzymatic digestion buffer: Hank's Balanced Salt Solution [HBSS] supplemented with 600 U/mL Collagenase II and 60 U/mL DNase I (Worthington). Afterwards, samples were digested for 30 min at 37°C in an ellipsoid shaker, and then further mechanically dissociated with GentleMACS (Miltenyi Biotec). Cell suspension was filtered through a 40 μm cell strainer. Finally, cells were washed and re-suspended in PBS, cell count estimated with hemocymeter counting and then concentration adjusted to 10^6^-10^7^ cells/150–200 μL of PBS. Viability of cells was determined using a Live/Dead staining of nitrogenated products (Thermofisher). Prior to immunostaining, samples were treated for 5 min with unconjugated Fc Receptor Binding Inhibitor (αCD16/32 for mice, Biolegend; and pan-Fc blocker for human samples, eBioscience). Surface immunostaining was performed using standard protocols, concentrations and times of incubation suggested by vendors of the fluorochrome-labeled antibodies (eBiosciences, BD Biosciences and BioLegend). For intracellular cytokine staining, cells were fixed and then permeabilized with appropriated nuclear staining reagents from eBiosciences. Experimental FACS data were acquired with an LSR II (BD Biosciences) or ImageStream MK-II (Millipore). Gates were stablished based on proper Fluorescent Minus One (FMO) Controls for markers with incomplete separation (Supplementary Figure 8). For FACS sorting the processing steps were identical. Heart ILCs CD45^+^CD31^neg^Lineage^neg^CD90^+^ were sorted using a FACSAria II Cell Sorter (BD Biosciences) and collected in HBSS + 10% FBS. Data were analyzed with FlowJo v10.4 (Tree Star), tSNE algorithm embedded in FlowJo v10.4 (Tree Star) and for Imaging Flow cytometry we used Ideas v6.0 (Millipore). tSNE analyses were performed on concatenates of all samples belonging to determinate experimental group, using 1,000 iterations with the following parameters: Perplexity 20, Theta 0.5, and Learning rate of 200.

### Fluorescent Antibodies

Anti-human Abs: GATA3 (PE-Cy7), Tbet (PerCP-Cy5.5), CD56 (PE-Texas Red), IL23R (PE), CD3, TCRαβ, TCRγδ, CD19, CD20, CD1a, CD11b, CD11c, CD123, BDCA2, CD14, FcεR1a (FITC), CD127 (BV711), NKp44 (BV605), CRTH2 (BV421), CD117 (APC-Cy7), CD4 (AF700), Rorγt (APC), CD45 (BUV395).

Anti-mouse Abs: PLZF (PE-Cy7), GATA3 (PerCP-Cy5.5), CD45 (PE-Texas Red), CD3, CD8, CD5, TCRβ, CD19, CD11b, CD11c, GR1, TER119, CD31, FcεRIα (FITC), Ki67 (BV605), ST2 (BV421), KLRG1 (APC-Cy7), CD4 (AF700), CD90 (BUV395), CD11b (APC), NKp46 (BV711), IL25R (PE).

### *In vitro* Culture

For *in vitro* experiments, hearts from 10 naïve Balb/cJ WT mice were processed and pooled. After sorting cardiac ILCs, cells were washed and resuspended in standard lymphoid cell-appropriated media: RPMI + L-glutamate + non-essential aminoacids + sodium piruvate + penicillin/streptomycin. Cells were placed in round bottom 96-well plates in a concentration of ≈600–800 cells/well/200 μL. Recombinant IL-2, IL-7, IL-33, IL-12, IL-23, IL-1β (Biolegend) were added at a concentration of 20 ng/mL. At the end of the pre-established time of culture, cells were spin down (300 g × 8 min), harvested, washed and stained for FACS as described above. Supernatants were collected for ELISA analysis (IL-5, IL-13, IL-17A, TNFα, IFNγ, R&D Systems).

### Adoptive Transfer of ILCs

Bone marrow cells were obtained by flushing mechanically the content of the femurs of Id2 reporter mice. Both femurs were harvested for each mouse, and 10 mice pooled for each transfer experiment. In brief, both femoral epiphysis were surgically removed after euthanasia of a donor mice. Then, using a 22G needle and a syringe loaded with 4 mL of PBS, the bone marrow was flushed into a 15 mL conical tube. After washing and standard treatment with ACK buffer, the single cell suspension was handled as described for FACS staining. Then, using FACSAria II, the CD45^+^Lineage^negative^Sca-1^+^Id2^+^ ILCPs were FACS sorted as described above. Finally, cells were resuspended at a concentration of 10^4^ ILCPs (CD45^+^Lin^negative^Sca-1^+^Id2^+^) per 200 μL of RPMI and injected intravenously in mice retro-orbitally.

### Parabiosis Surgery

Mice were anesthetized with inhaled isoflurane, 4.0–5.0%. Maintenance anesthesia was kept during surgery with intramuscular injections of ketamine (80 mg/kg) and xylazine (16 mg/kg). For complementary analgesia buprenorphine (0.1 mg/kg) was intraperitoneally injected concurrent to initial analgesia, and 12 h postoperatively. Longitudinal incisions in the skin and subcutaneous tissues were made through the skin starting from the elbow joint and extended down to the knee joint. In order to promote skin anastomosis, a continuous 5-0 absorbable Vicryl suture was also used through the muscular layer to connect the pairs. Non-absorbable 4-0 discontinuous sutures were made to attach corresponding subcutaneous tissues between parabionts. Surgical stapler was used to connect the skins of the pairs. Baytril (Enrofloxacin) was used upon the completion of the procedure as antibiotic prophylaxis. All parabiosis surgeries were done by one surgeon (JS). Animals were provided with moistened chow and gel food diet supplement every other day until sacrifice on days 19–20 after the parabiosis. Mice were daily evaluated for signs of pain and discomfort. In the set of experiments reported in this study, no additional analgesia doses were required. The establishment of mixed chimerism was evaluated on day 14 after the surgery in peripheral blood by FACS using CD45.1 and CD45.2 immunostaining of PBMCs.

### Statistical Analyses

Comparison between groups was estimated as follows: (a) two independent groups using Student t, (b) three or more independent experimental groups using one-way ANOVA plus Bonferroni's *post-test*, (c) two experimental groups in respect to a single control group using Dunnetts's test. Significance of numerical correlations was calculated with Pearson r of best-fitting mathematical functions. Shapiro-Wilk test was used to verify Gaussian distribution prior to further statistical analysis. We used non-parametric U-Mann Whitney for comparison of histological scores between 2 groups. In every case α = 0.05 and β = 0.20 (Potency = 80%) were used as thresholds to estimate significance. All calculations were made using GraphPad Prism v6.0.

## Author Contributions

WB-B conceptualization, designed experiments, performed the experiments, analyzed data, wrote the manuscript, funding acquisition. GC, XH, MT, HC, and GD performed some experiments, gathered clinical data. HT conceptualization, obtained human cardiac biopsy samples. JS and DH helped to designed and perform parabiosis experiments. DN conceptualization, gathered clinical data. DČ conceptualization, designed experiments, analyzed data, wrote the manuscript, main funding acquisition. All authors approved the manuscript.

### Conflict of Interest Statement

The authors declare that the research was conducted in the absence of any commercial or financial relationships that could be construed as a potential conflict of interest.

## Supplementary Material

Supplementary Figure 1 Flow cytometry gating strategy for human ILCs. Basic gating strategy of human cardiac ILCs. FSC and SSC parameters allowed an initial morphologic gate and exclusion of doublets. Live/Dead Aqua dye was used to exclude dead cells and cells with incompetent membrane. ILCs were defined CD45^+^Lineage^neg^CD127^+^. The Tbet^neg^Rorγ^neg^ population was also negative for NKp44 and IL-23R.

Supplementary Figure 2 Comprehensive analysis of cardiac ILCs, including NK cells and non-cytotoxic ILC population. **(A)** Gating strategy in which CD11b was excluded of the Lineage cocktail. NK cells were CD45^+^CD11b^+^CD56^+^ CD127^neg^ and predominantly NKp44^neg^. Non-cytotoxic ILCs were defined as Lineage^neg^CD11b^neg^CD56^neg^CD127^+^. **(B)** Bar graphs showing the proportion of CD45^+^Lineage^neg^ cells represented by NK cells and the non-cytotoxic ILC subsets.

Supplementary Figure 3 Analysis of circulating ILCs in peripheral blood. Basic gating strategy used to analyze non-cytotoxic ILCs and NK cells in PBMCs. CD11b was removed from the Lineage cocktail. Activated NK cells were defined as NKp44^+^CD56^+^Tbet^+^, whereas non-cytotoxic ILCs were CD127^+^NKp44^neg^. ILC3s (red) were IL23R+Tbetint, ILC1 (blue) were Tbet+ and ILC2s (orange) were defined based on CRTH2 expression (FMO control is shown in dashed histogram).

Supplementary Figure 4 Expansion of absolute number of ILC2s during MI and EAM. **(A)** Representative H&E ventricular histology images of EAM and mock immunized mice, and histology scores. **(B)** Bar graphs showing the similarity of ILC compartment composition in naïve, mock immunized and sham surgery controls. **(C)** Flow cytometry analysis of Rorγt and FcεR1a in NKp46^+^CD90^+^ ILCs. **(D)** Flow cytometry plots showing cKit and IL25R expression in total heart's ILCs population. **(E)** Absolute number of murine cardiac ILC2s in control, MI and EAM hearts. **(F)** Absolute number of undifferentiated ILC in control, MI, EAM hearts. Flow plots show concatenates of representative examples of 1 of 3 independent experiments, where *n* = 5 for naïve controls and EAM, *n* = 4 for MI in this experiment, and *n* = 2–3 mock and sham. Bar graphs shows Mean and SD. Statistics were calculated using Dunnett's test. ^*^*P* < 0.05. ^**^ *P* < 0.01.

Supplementary Figure 5 Comprehensive analysis of murine ILC compartment including NK cells and non-cytotoxic ILCs. **(A)** Basic gating strategy followed to analyze murine cardiac ILCs and NK cells. CD11b and CD3 were placed in independent channels out of the Lineage cocktail. Classic NK cells were CD11b^+^CD90^neg^NKp46^+^CD122^+^. CD11b^neg^ NK cells were CD90^+^NKp46^+^CD122^+^. Non-cytotoxic were defined ILC1s (blue) as Tbet^+^, ILC2s (gray) as ST2^+^KLRG1^+^, ILC3s (transparent red) as Rorγt^+^. Undifferentiated ILCs (transparent orange) as ILCs negative for all ILC type-specific markers. **(B)** Bar graphs showing the proportion of total leukocytes (CD45^+^ cells) represented by the NK and non-cytotoxic ILC subsets.

Supplementary Figure 6 tSNE analysis of cardiac fibroblast population shows restriction of IL-33^+^ cells to Sca-1^+^ cardiac fibroblast cluster. tSNE plots of cardiac fibroblasts and IL-33 production in naïve hearts, MI and EAM. It shows Sca-1^neg^ cells in gray, Sca-1^+^ in orange, and IL-33^+^ events in green. Majority of IL-33^+^ events (green) overlap with Sca-1^+^ cluster (orange).

Supplementary Figure 7 *In vitro* responses to IL-23 are comparable to IL-1β and *in vitro* culture of ST2+ non-cytotoxic ILCs. **(A–C)** Flow plots showing phenotypic differentiation *in vitro* of naïve heart ILCs under control IL-2+IL-7+IL-1β differentiation condition. **(D,E)** Bar graphs comparing cardiac ILCs differentiation under control, IL-23- and IL-1β-inducing type 3 conditions. **(F)** ELISA results showing the cytokine production profile of cardiac ILCs under control, IL-23- and IL-1β-inducing type 3 conditions. **(G)** Flow cytometry plots gated on live cells, showing the cellularity retrieved after a 6-day culture of ST2+ non-cytotoxic ILCs in IL-2+IL-7+IL-33 conditions. Flow cytometry plots show median representative examples. Graphics show results of 1 of 2–4 independent experiments, each one made in triplicates for each condition. Bar graphs shows Mean and SD. Statistics calculated with one-way ANOVA and Bonferroni *post-test*.

Supplementary Figure 8 Fluorescence Minus One (FMO) controls. Representative examples of histogram overlay of FMO controls (gray) with stained cardiac ILCs (open red histogram) used to set flow cytometry gates in human **(A)** and mouse **(B)** experiments.

## Abbreviations

CCR-: C-C chemokine receptor
CHILP: common helper-like innate lymphoid progenitor
cKit: proto-oncogene tyrosine-protein kinase Kit (synonym of CD117 and mast/stem cell growth factor receptor)
CD: cluster of differentiation
CRTH2, chemoattractant Receptor-homologous molecule expressed on T-Helper type 2 cells (synonymous of DP2: Prostaglandin D_2_ receptor 2)
EAM: experimental autoimmune myocarditis
GATA3: G-A-T-A sequence-recognizing transcription factor 3
Id2: DNA-binding protein inhibitor 2
i-ILC: inflammatory innate lymphoid cell
ILC: innate lymphoid cell
ILCP: innate lymphoid cell progenitor
KLRG1: killer cell lectin-like receptor subfamily G member 1
LVAD: left ventricle assist device
MFI: geometric mean fluorescence intensity
MI: myocardial infarction
NK: natural killer
PBMC: peripheral blood mononuclear cell
PLZF: pro-myelocytic leukemia zinc finger protein
Rorγt: retinoic acid receptor alpha-related orphan receptor gamma
Tbet: T-box transcription factor TBX21
tSNE: t-distributed stochastic neighbor embedding
γc: IL-2 receptor common gamma chain.
