# Supplementary figures and images for: Non-cytotoxic Cardiac Innate Lymphoid Cells Are a Resident and Quiescent Type 2-Commited Population
Source: Front Immunol. 2019 Mar 29;10:634. doi: 10.3389/fimmu.2019.00634 (PMC6450181; doi:10.3389/fimmu.2019.00634)

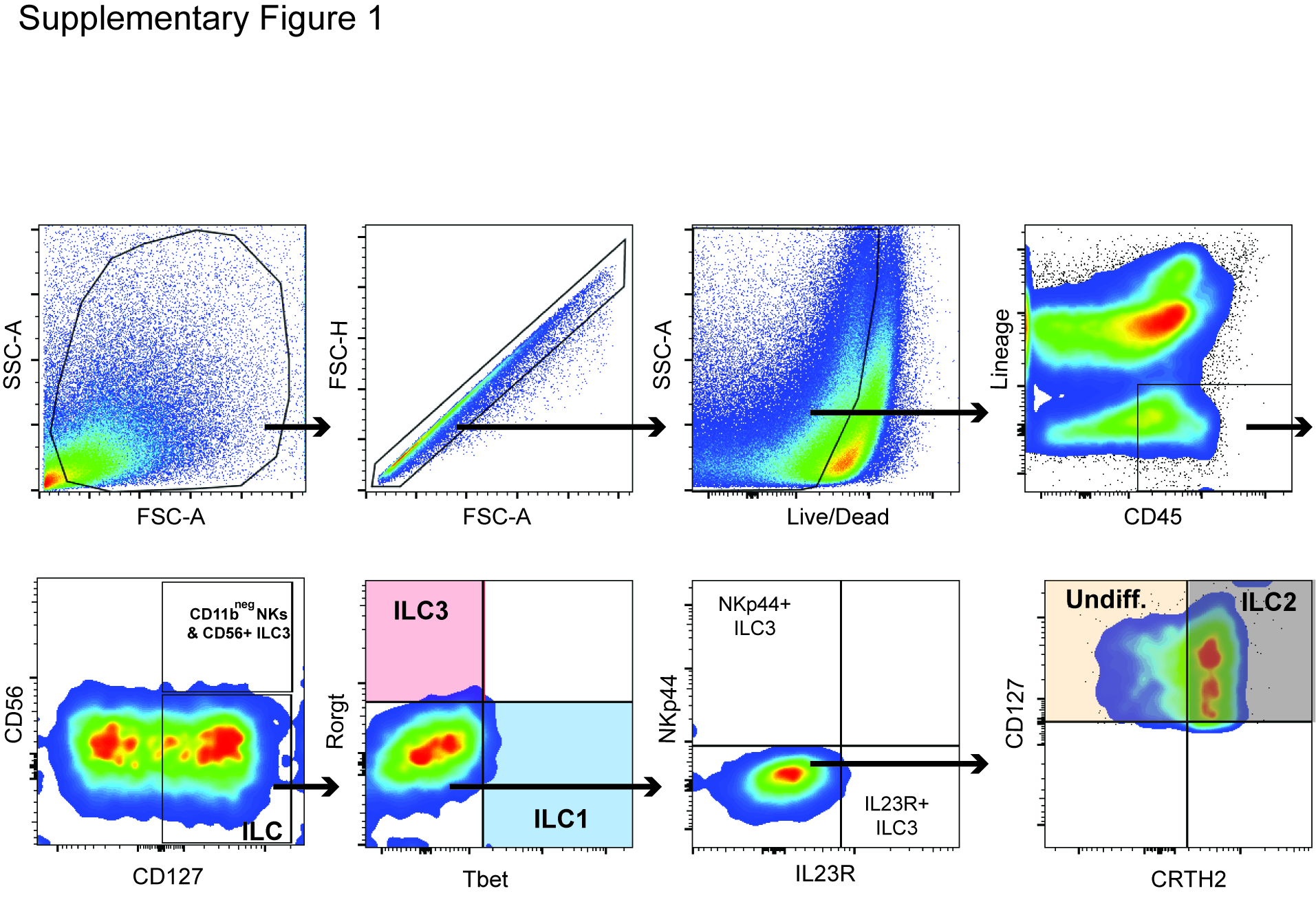

Supplement: Supplementary Figure 1 — Flow cytometry gating strategy for human ILCs. Basic gating strategy of human cardiac ILCs. FSC and SSC parameters allowed an initial morphologic gate and exclusion of doublets. Live/Dead Aqua dye was used to exclude dead cells and cells with incompetent membrane. ILCs were defined CD45+LineagenegCD127+. The TbetnegRorγneg population was also negative for NKp44 and IL-23R. [file Image_1.TIF]

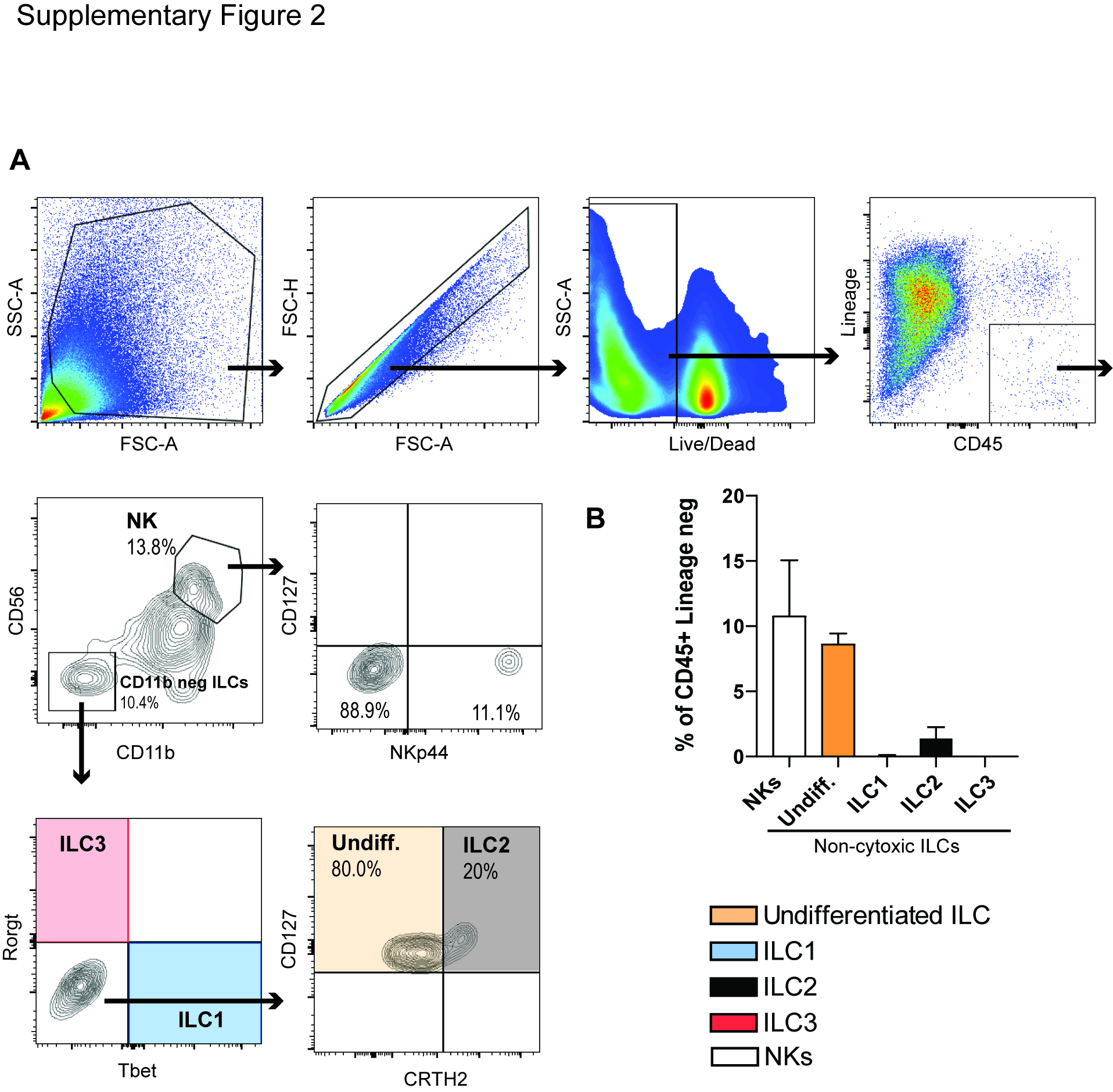

Supplement: Supplementary Figure 2 — Comprehensive analysis of cardiac ILCs, including NK cells and non-cytotoxic ILC population. (A) Gating strategy in which CD11b was excluded of the Lineage cocktail. NK cells were CD45+CD11b+CD56+ CD127neg and predominantly NKp44neg. Non-cytotoxic ILCs were defined as LineagenegCD11bnegCD56negCD127+. (B) Bar graphs showing the proportion of CD45+Lineageneg cells represented by NK cells and the non-cytotoxic ILC subsets. [file Image_2.TIF]

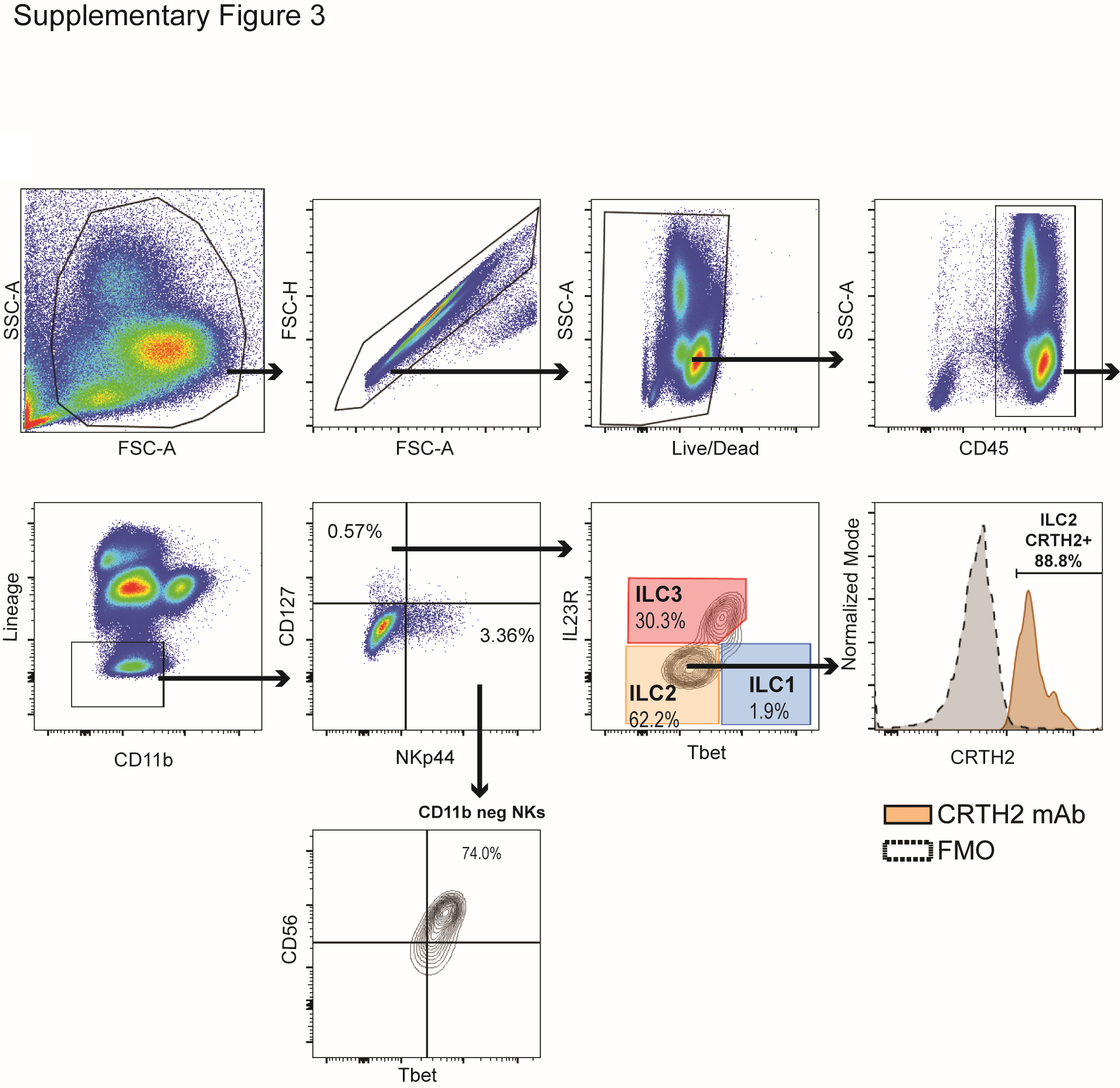

Supplement: Supplementary Figure 3 — Analysis of circulating ILCs in peripheral blood. Basic gating strategy used to analyze non-cytotoxic ILCs and NK cells in PBMCs. CD11b was removed from the Lineage cocktail. Activated NK cells were defined as NKp44+CD56+Tbet+, whereas non-cytotoxic ILCs were CD127+NKp44neg. ILC3s (red) were IL23R+Tbetint, ILC1 (blue) were Tbet+ and ILC2s (orange) were defined based on CRTH2 expression (FMO control is shown in dashed histogram). [file Image_3.tif]

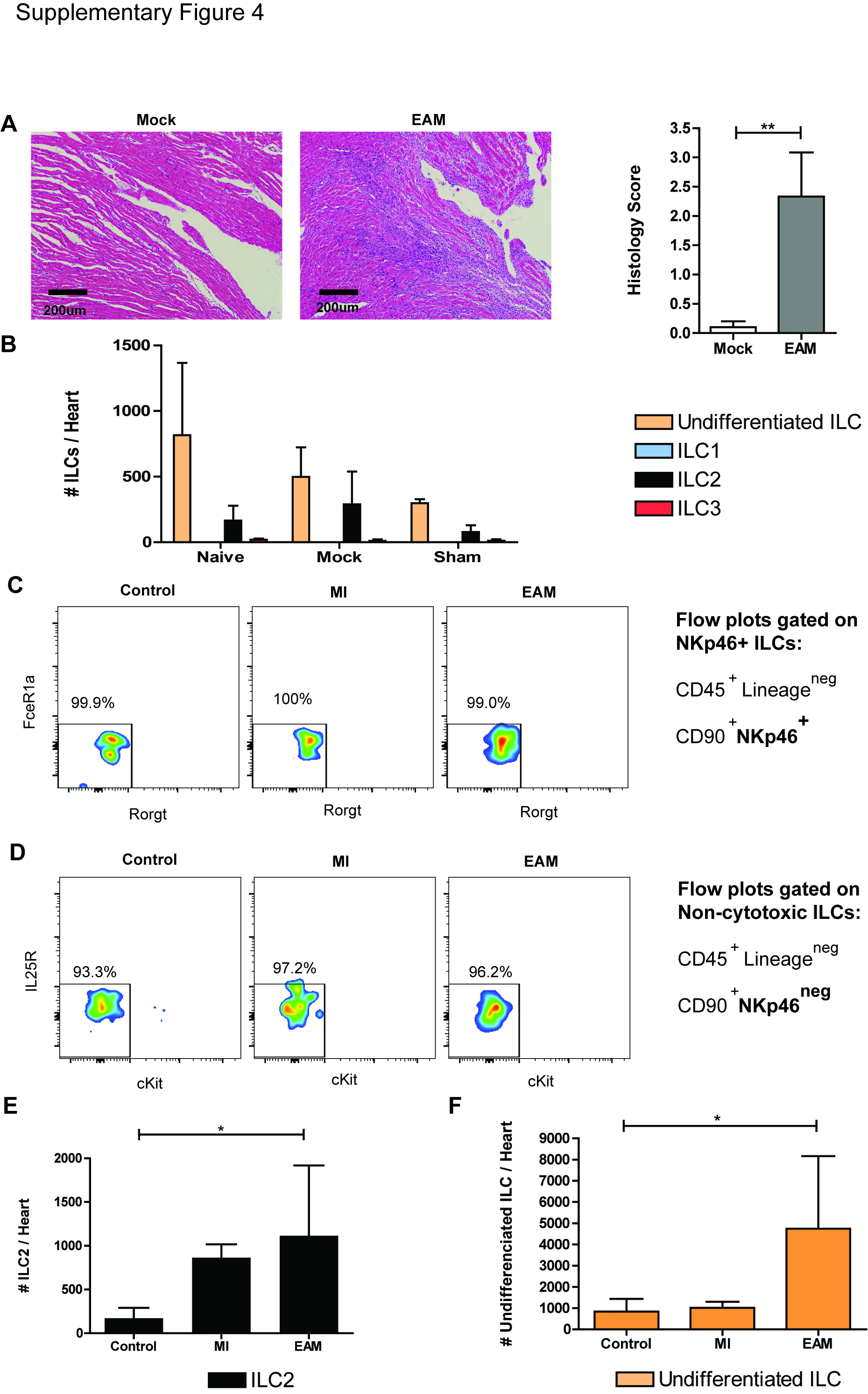

Supplement: Supplementary Figure 4 — Expansion of absolute number of ILC2s during MI and EAM. (A) Representative H&E ventricular histology images of EAM and mock immunized mice, and histology scores. (B) Bar graphs showing the similarity of ILC compartment composition in naïve, mock immunized and sham surgery controls. (C) Flow cytometry analysis of Rorγt and FcεR1a in NKp46+CD90+ ILCs. (D) Flow cytometry plots showing cKit and IL25R expression in total heart's ILCs population. (E) Absolute number of murine cardiac ILC2s in control, MI and EAM hearts. (F) Absolute number of undifferentiated ILC in control, MI, EAM hearts. Flow plots show concatenates of representative examples of 1 of 3 independent experiments, where n = 5 for naïve controls and EAM, n = 4 for MI in this experiment, and n = 2–3 mock and sham. Bar graphs shows Mean and SD. Statistics were calculated using Dunnett's test. *P < 0.05. ** P < 0.01. [file Image_4.TIF]

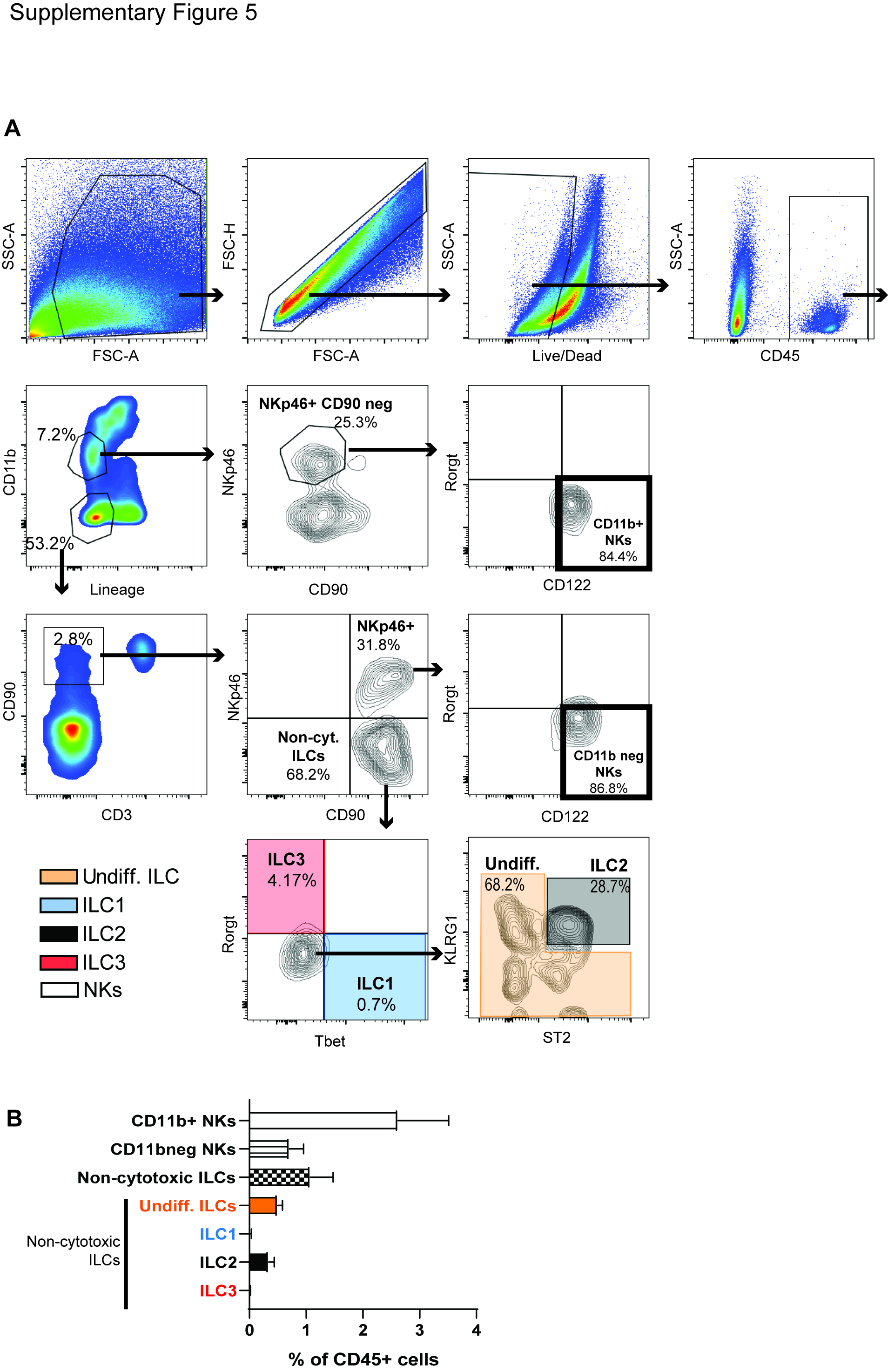

Supplement: Supplementary Figure 5 — Comprehensive analysis of murine ILC compartment including NK cells and non-cytotoxic ILCs. (A) Basic gating strategy followed to analyze murine cardiac ILCs and NK cells. CD11b and CD3 were placed in independent channels out of the Lineage cocktail. Classic NK cells were CD11b+CD90negNKp46+CD122+. CD11bneg NK cells were CD90+NKp46+CD122+. Non-cytotoxic were defined ILC1s (blue) as Tbet+, ILC2s (gray) as ST2+KLRG1+, ILC3s (transparent red) as Rorγt+. Undifferentiated ILCs (transparent orange) as ILCs negative for all ILC type-specific markers. (B) Bar graphs showing the proportion of total leukocytes (CD45+ cells) represented by the NK and non-cytotoxic ILC subsets. [file Image_5.TIF]

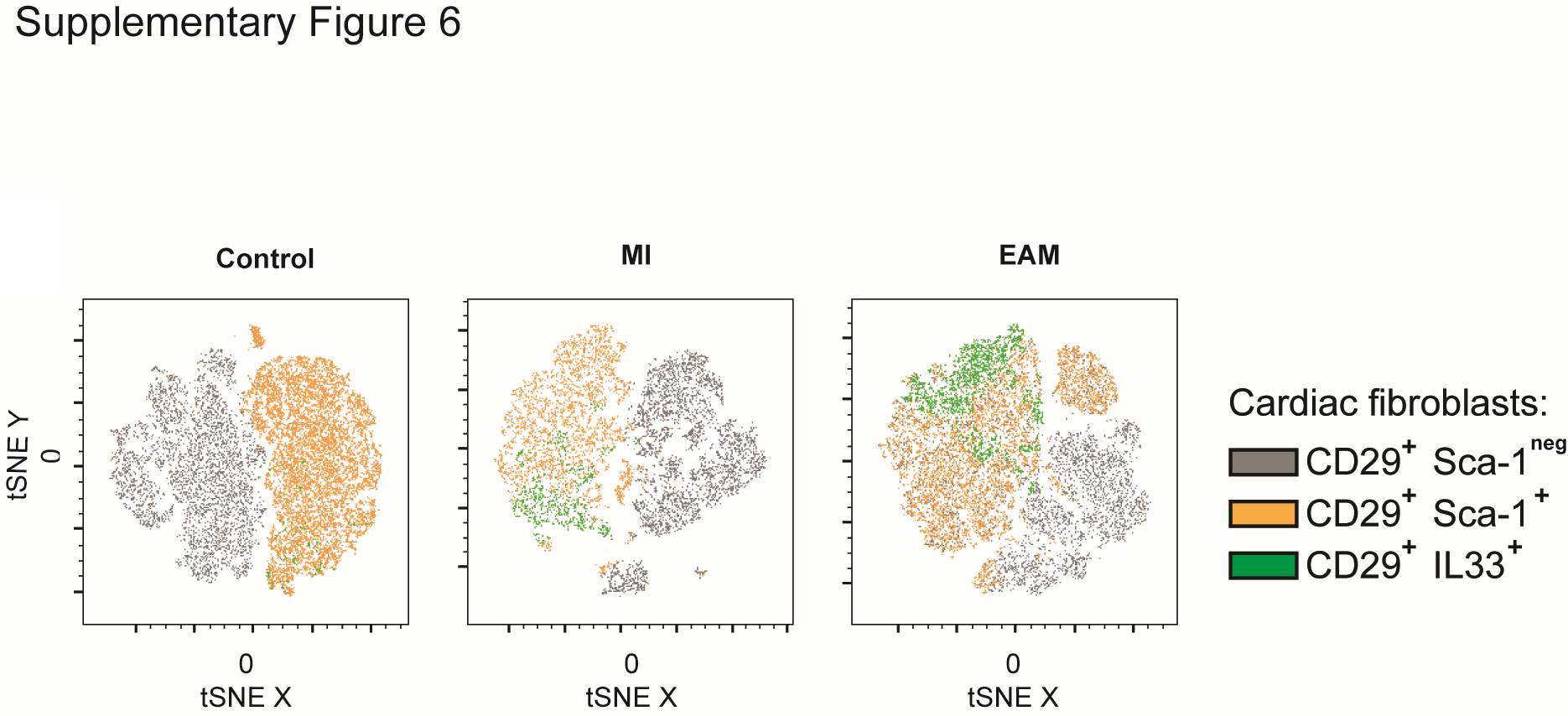

Supplement: Supplementary Figure 6 — tSNE analysis of cardiac fibroblast population shows restriction of IL-33+ cells to Sca-1+ cardiac fibroblast cluster. tSNE plots of cardiac fibroblasts and IL-33 production in naïve hearts, MI and EAM. It shows Sca-1neg cells in gray, Sca-1+ in orange, and IL-33+ events in green. Majority of IL-33+ events (green) overlap with Sca-1+ cluster (orange). [file Image_6.tif]

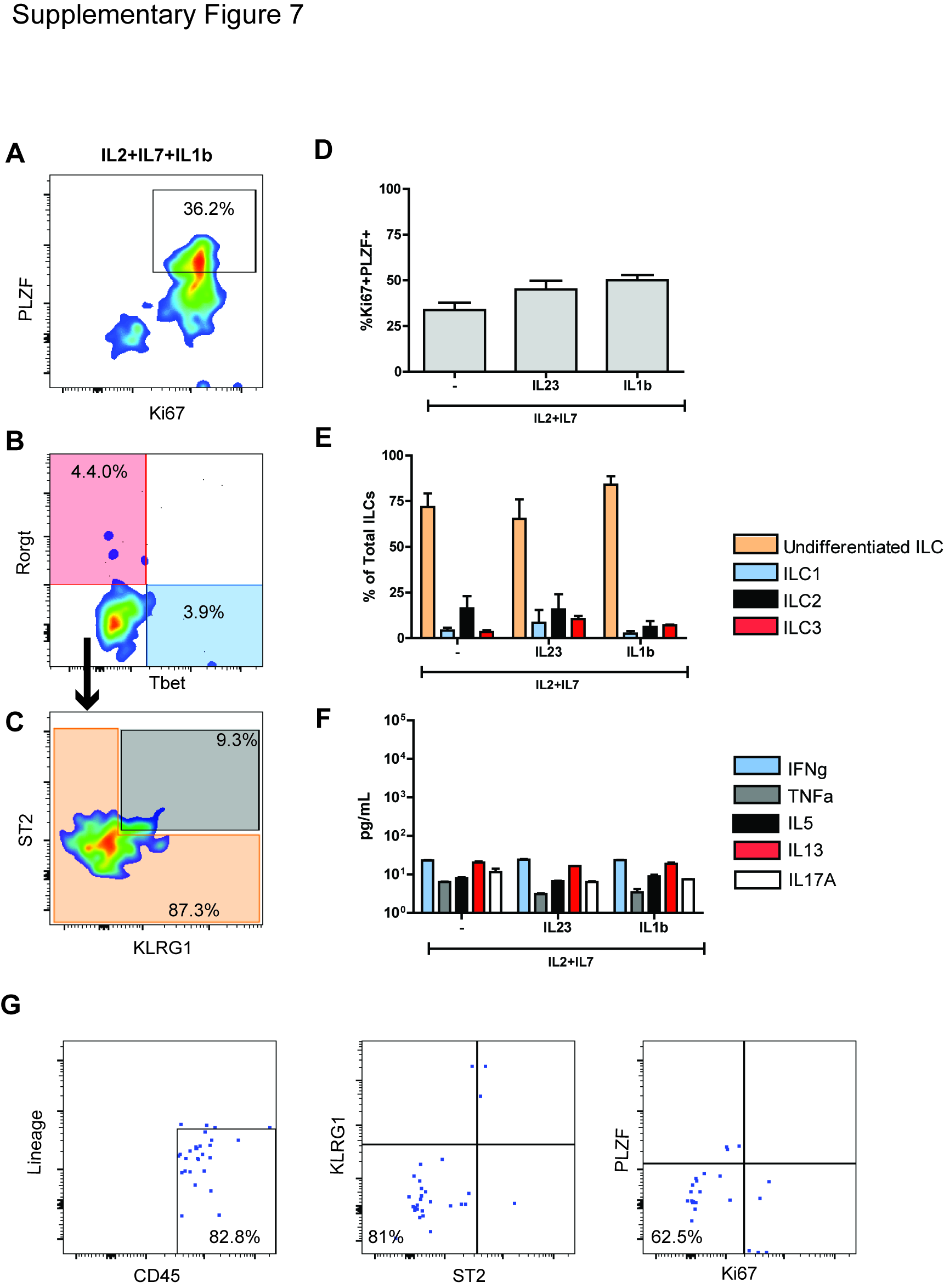

Supplement: Supplementary Figure 7 — In vitro responses to IL-23 are comparable to IL-1β and in vitro culture of ST2+ non-cytotoxic ILCs. (A–C) Flow plots showing phenotypic differentiation in vitro of naïve heart ILCs under control IL-2+IL-7+IL-1β differentiation condition. (D,E) Bar graphs comparing cardiac ILCs differentiation under control, IL-23- and IL-1β-inducing type 3 conditions. (F) ELISA results showing the cytokine production profile of cardiac ILCs under control, IL-23- and IL-1β-inducing type 3 conditions. (G) Flow cytometry plots gated on live cells, showing the cellularity retrieved after a 6-day culture of ST2+ non-cytotoxic ILCs in IL-2+IL-7+IL-33 conditions. Flow cytometry plots show median representative examples. Graphics show results of 1 of 2–4 independent experiments, each one made in triplicates for each condition. Bar graphs shows Mean and SD. Statistics calculated with one-way ANOVA and Bonferroni post-test. [file Image_7.TIF]

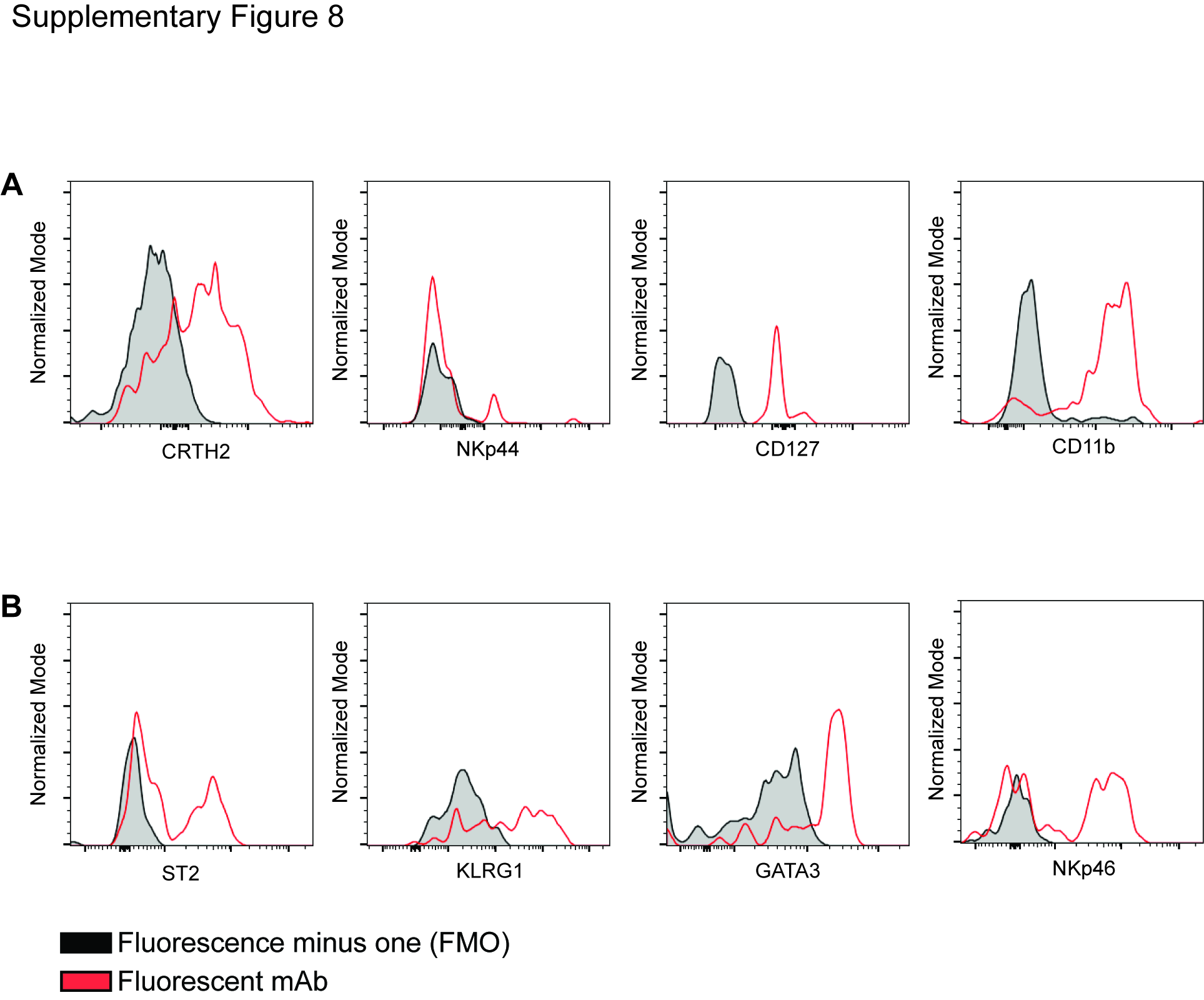

Supplement: Supplementary Figure 8 — Fluorescence Minus One (FMO) controls. Representative examples of histogram overlay of FMO controls (gray) with stained cardiac ILCs (open red histogram) used to set flow cytometry gates in human (A) and mouse (B) experiments. [file Image_8.TIF]
